# Structural insights into the broad protection against H1 influenza viruses by a computationally optimized hemagglutinin vaccine

**DOI:** 10.1038/s42003-023-04793-3

**Published:** 2023-04-25

**Authors:** John V. Dzimianski, Julianna Han, Giuseppe A. Sautto, Sara M. O’Rourke, Joseph M. Cruz, Spencer R. Pierce, Jeffrey W. Ecker, Michael A. Carlock, Kaito A. Nagashima, Jarrod J. Mousa, Ted M. Ross, Andrew B. Ward, Rebecca M. DuBois

**Affiliations:** 1 Department of Biomolecular Engineering, University of California Santa Cruz, Santa Cruz, CA USA; 2 Department of Integrative Structural and Computational Biology, The Scripps Research Institute, La Jolla, CA USA; 3 Florida Research and Innovation Center, Cleveland Clinic, Port Saint Lucie, FL USA; 4 Center for Vaccines and Immunology, College of Veterinary Medicine, University of Georgia, Athens, GA USA; 5 Department of Infectious Diseases, College of Veterinary Medicine, University of Georgia, Athens, GA USA; 6 Department of Biochemistry and Molecular Biology, Franklin College of Arts and Sciences, University of Georgia, Athens, GA USA

**Keywords:** Vaccines, Structural biology

## Abstract

Influenza virus poses an ongoing human health threat with pandemic potential. Due to mutations in circulating strains, formulating effective vaccines remains a challenge. The use of computationally optimized broadly reactive antigen (COBRA) hemagglutinin (HA) proteins is a promising vaccine strategy to protect against a wide range of current and future influenza viruses. Though effective in preclinical studies, the mechanistic basis driving the broad reactivity of COBRA proteins remains to be elucidated. Here, we report the crystal structure of the COBRA HA termed P1 and identify antigenic and glycosylation properties that contribute to its immunogenicity. We further report the cryo-EM structure of the P1-elicited broadly neutralizing antibody 1F8 bound to COBRA P1, revealing 1F8 to recognize an atypical receptor binding site epitope via an unexpected mode of binding.

## Abstract (web summary)

Structural studies of a computationally optimized broadly reactive antigen hemagglutinin in complex with a broadly neutralizing antibody reveal its immunogenic properties and provide insights into flu vaccine design.

## Introduction

Influenza remains an ongoing public health concern. Despite advances in vaccine technology, formulating broadly effective influenza vaccines remains a challenge. Due to the multiplicity of endemic influenza strains as well as virus evolution through point mutations (“antigenic drift”), annual vaccine efficacy ranges from 10 to 60%^1^. In addition to the annual burden posed by seasonal infections, influenza also possesses high pandemic potential with four historic pandemics in 1918, 1957–1958, 1968, and 2009^2^. These factors have led the National Institute of Allergy and Infectious Diseases (NIAID) to prioritize the research and development of more effective vaccines^3^.

One approach to attain more broadly protective vaccines is the use of computationally optimized broadly reactive antigens (COBRAs). The COBRA approach utilizes known sequence information to generate composite proteins representing a broad swath of viruses. Input sequences from a desired antigen target are subjected to an iterative series of sequence alignments to generate primary, secondary, and tertiary (or more) consensus sequences that converge to a final optimized antigen representing both conserved and divergent features within the design space^4^. Using this method, COBRA hemagglutinin (HA) proteins have been generated that are more broadly protective than wildtype proteins when used as vaccine immunogens in preclinical models^4–7^. In some cases, these COBRA HAs are protective not only against viruses that postdate the time-range of the input design sequences, but also emerging strains^8^, suggesting that antigens designed by this method elicit immune responses that are resilient to antigenic drift^9^.

**Fig. 1:**
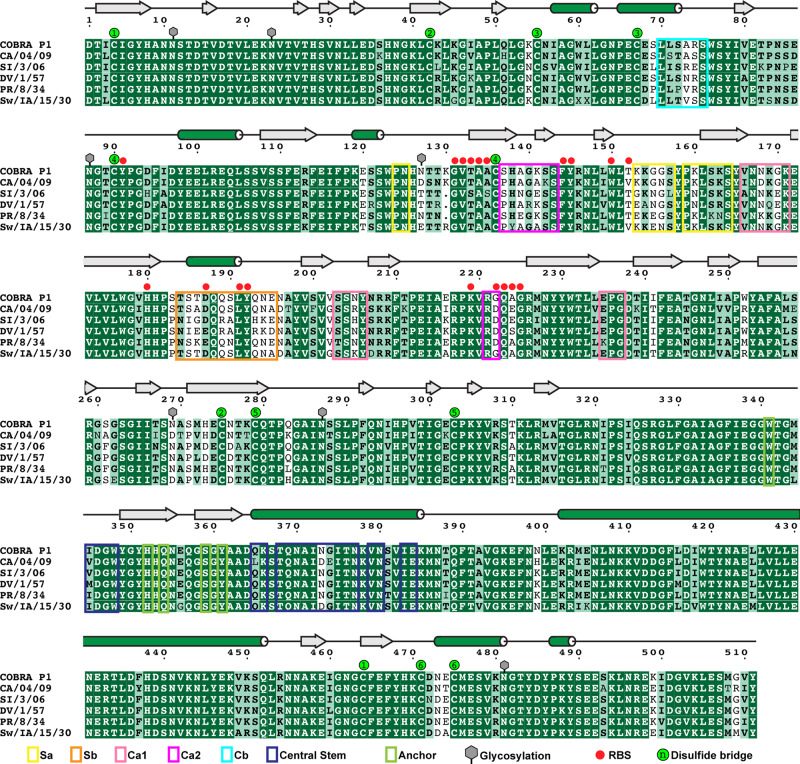
Sequence alignment of HA proteins. The ectodomain of mature HA proteins for COBRA P1, A/California/04/09 (CA/04/09; Accession number ACP41105.1), A/Solomon Islands/3/2006 (SI/3/06; Accession number ABU99109.1), A/Denver/1/1957 (DV/1/57; Accession number ABD15258.1), A/Puerto Rico/8/1934 (PR/8/34; Accession number ADX99484.1) and A/Swine/Iowa/15/1930 (Sw/IA/15/30; Accession number Q9WCD9.1) were aligned with CLUSTAL OMEGA^77^ and manually checked. The initial graphic was generated with the ESPript 3.0 server^78^ and annotated in Adobe Illustrator. The secondary structure assignments based on a DSSP analysis^79^ of the crystal structure are denoted as silver block arrows for β-sheets and green cylinders for α-helices. H1N1pdm residue numbering is used^58^. Antigenic regions for Sa, Sb, Ca1, Ca2, Cb, Central Stem, and Anchor are shown using yellow, orange, magenta, purple, sky blue, royal blue, and bright green colored boxes. RBS, receptor binding site (red circles). N-linked glycosylation observed in the crystal structure is indicated by gray hexagons. Numbered green circles denote cysteines paired together in disulfide binds.

Among the effective HA candidates designed for H1 influenza viruses is the COBRA HA termed P1 (Fig. 1). P1 was constructed using a combination of human H1N1 sequences spanning the years 1933–1957 and 2009–2011, along with swine sequences from 1931 to 1998^10^. P1 elicits protective immunity in mice to a broad range of H1N1 viruses, including historic strains, swine strains, pandemic strains such as A/California/04/2009 (CA/04/09) and A/California/07/2009 (CA/07/09), and recently emerged strains like A/Brisbane/02/2018, A/Guangdong-Maonan/SWL1536/2019, and G4^7,8,10–12^. Specifically, P1 vaccination in mice results in the production of a broadly neutralizing antibody response, including the broadly reactive, head targeting monoclonal antibody 1F8^8,11^. Overall, these characteristics of P1 and similar antigens highlight the great potential of COBRAs to address current needs in vaccine development.

Although P1 elicits broad protection in mice against diverse H1 influenza viruses, the mechanistic details undergirding its breadth remain to be fully elucidated. While comparative immunological studies have inferred some antigenic features that play a role in eliciting a broadly reactive antibody response, the exact structural details are unknown^13^. In addition, the epitope(s) targeted by P1-produced broadly neutralizing antibodies, such as 1F8, have not been identified. To elucidate these details, we determined the structures of the COBRA P1 alone and in complex with 1F8. The 3.0 Å crystal structure of COBRA P1 reveals antigenic features and an atypical glycosylation site that influence the immunogenicity of the HA head domain. Using cryo-EM, we solved a 3.1 Å structure of 1F8 bound to P1, revealing 1F8 to be a receptor binding site (RBS) targeting antibody with a unique mode of binding relative to published antibodies. These data provide fresh insights into how COBRAs elicit broadly protective immunity that can inform future rational vaccine design.

## Results

### COBRA P1 HA forms a structurally intact prefusion trimer

**Fig. 2:**
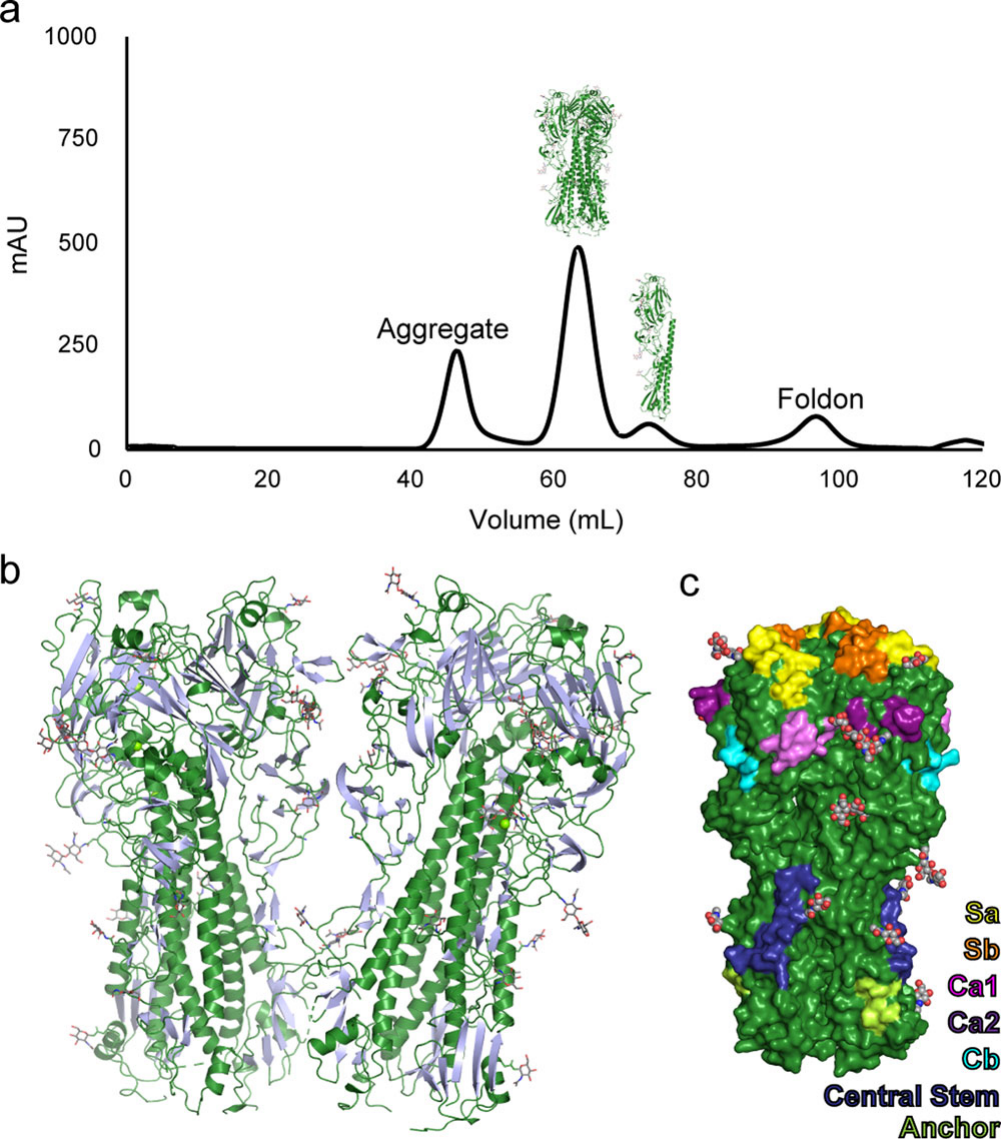
Purification and structural determination of COBRA P1. **a** Size exclusion trace of COBRA P1 following thrombin digestion to release the T4 fibritin Foldon trimerization domain and affinity tags. Trimeric HA formed the major peak, with aggregate and monomeric HA forming minor species. **b** The asymmetric unit of the crystal structure consisting of two trimers, with β-strands colored silver and loops and helices in forest green. **c** Surface rendering of a single trimer. The major head antigenic sites are shown in yellow, orange, magenta, purple, and sky blue for Sa, Sb, Ca1, Ca2, and Cb, respectively. Residues comprising the Central Stem and Anchor epitopes are shown in royal blue and bright green. Carbon and oxygen atoms in glycans are colored as gray and red spheres.

Initial attempts to solve a structure with mammalian expressed COBRA P1 HA containing a Foldon trimerization domain and affinity tags failed to yield diffracting crystals. To obtain a more optimal sample, the P1 gene was cloned into a vector for baculovirus-mediated expression and secretion in insect cells to generate a protein product with simpler glycosylation. In addition, a thrombin cleavage site was added to enable the removal of the flexible Foldon domain and affinity tags from the HA protein. Following affinity purification and digestion to release the tags, the majority of P1 protein product remained trimeric based on size exclusion chromatography, suggesting it retains structural integrity even in the absence of the stabilizing trimerization domain (Fig. 2a and Supplementary Fig. 1).

The trimeric P1 protein produced high quality crystals that resulted in a 3.0 Å-resolution structure (Fig. 2b, Supplementary Fig. 2, Table 1). The overall structure adopts a classical HA fold in the prefusion HA0 conformation, with tightly packed α-helices forming the stem and β-sheet rich regions comprising the head domain. Comparison with native HA sequences shows that the classical antigenic sites are surface-displayed and glycosylation modifications are present at the predicted motifs (Fig. 1 and Fig. 2b, c). Overall, the COBRA P1 HA exhibits native-like structural characteristics, confirming the structural integrity of these computationally designed proteins.

**Table 1:** Data collection and refinement statistics (molecular replacement).

	P1 COBRA (PDB 7UYI)
Data collection	
Space group	C2_1_
Cell dimensions	
*a*, *b*, *c* (Å)	264.85, 77.56, 222.52
α, β, γ (°)	90.00, 93.77, 90.00
Resolution (Å)	46.75-3.00 (3.05-3.00)^a^
*R*_merge_	0.222 (1.435)
*I*/σ*I*	6.6 (1.7)
CC(1/2)	0.988 (0.429)
Completeness (%)	99.8 (97.7)
Redundancy	6.2 (5.8)
Refinement	
Resolution (Å)	46.75-3.00 (3.11-3.00)
No. reflections	90,750 (8898)
*R*_work_/*R*_free_	0.226 (0.270)
No. atoms	
Protein	22570
Ligand/ion	745
Water	23
*B*-factors	
Protein	79.80
Ligand/ion	104.30
Water	38.28
R.m.s. deviations	
Bond lengths (Å)	0.002
Bond angles (°)	0.48

^a^Values in parentheses are for highest-resolution shell.

### The structural features of immunodominant antigenic sites reflect immune protection

**Fig. 3:**
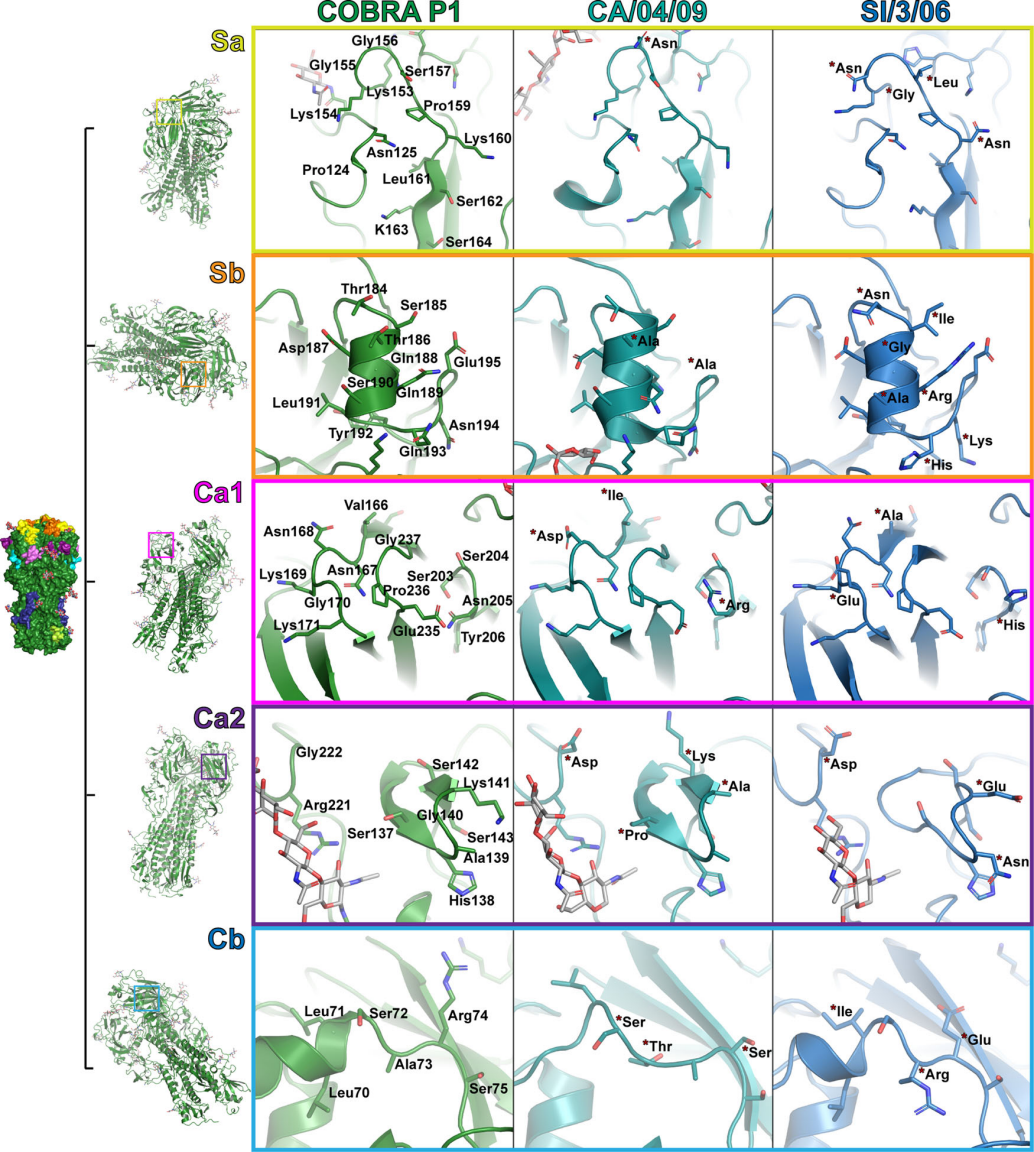
Structural features of HA head antigenic sites. Crystal structures of HA from CA/04/09 and SI/3/06 (PDB entries 3LZG and 6CF7) were aligned to COBRA P1. The residues forming the major head antigenic sites Sa, Sb, Ca1, Ca2, and Cb are shown as sticks. Residue names and numbering are shown for COBRA P1, with those that differ in CA/04/09 and SI/3/06 indicated by red asterisks with differing amino acid labels.

P1 elicits protective antibodies against historic influenza strains as well as pandemic strains, such as CA/04/09. It fails to protect, however, against some pre-pandemic seasonal strains such as A/Brisbane/59/2007 (Br/59/07) and Solomon Islands/3/2006 viruses (SI/3/06)^7,10^. Comparison of P1 to the CA/04/09 and SI/3/06 HA structures reveals these immunological patterns correlate with the similarity of the major head antigenic sites with those in P1 (Fig. 3 and Supplementary Fig. 3)^14,15^.

In the Sa site, CA/04/09 is almost identical to P1 with only a single residue change. In contrast, SI/03/06 contains four amino acid differences. Although the change in CA/04/09 from a glycine to an asparagine at residue 156 could impact the flexibility of the loop, the fundamental surface features of the site change very little. In SI/03/06, however, the changes include the loss of charged amino acids at positions 153 and 160 and the addition of a leucine at 157 that presents a more neutral and hydrophobic interface that may disrupt antibody binding. In a similar manner, the Sb site in CA/04/09 shows relatively small changes, with alanine residues substituted for threonine and glutamate at positions 186 and 195. While this involves the loss of polar and charged amino acids, respectively, it represents merely the loss of potential interacting sidechains without introducing additional disruptive effects in the binding interface. SI/3/06, on the other hand, possesses an isoleucine, arginine, and lysine in place of serine, glutamine, and asparagine at residues 185, 189, and 194, respectively, among the seven amino acid differences. These residues not only alter the electrostatic surface, but also present bulky side chains that would be expected to disrupt binding from antibodies targeting this site in P1.

In contrast to Sa and Sb, the Ca1, Ca2, and Cb sites all show a high degree of variability in both CA/04/09 and SI/3/06. Upon closer examination, however, there are subtle differences that may favor CA/04/09. In the Ca1 site, the biggest difference in CA/04/09 is an arginine rather than an asparagine residue at position 205, with isoleucine versus valine and aspartate versus asparagine resulting in residues with similar properties at positions 166 and 168. On the other hand, SI/03/06 contains a glutamate instead of a glycine at residue 170, resulting in a big change in shape and charge within the surface-exposed region of the antigenic site. In Ca2, both P1 and CA/04/09 prominently feature a lysine residue at positions 141 and 142, respectively, albeit in a flipped residue order. SI/03/06 instead contains a glutamate at residue 141, essentially switching the surface charge at this antigenic site. Finally, while both CA/04/09 and SI/3/06 HAs exhibit three amino acid differences in Cb, the changes in CA/04/09 are for small, polar amino acids whereas SI/3/06 includes the addition of an additional charged residue at position 73.

Examination of the highly conserved HA Central Stem and Anchor epitopes reveals similar sequences between P1 and historic and pandemic strains (Fig. 1)^16–18^. However, whereas P1 and SI/03/06 are nearly identical in the Stem epitope, some differences in CA/04/09, such as Asp373 and Glu374, may affect P1-elicited antibodies from targeting this site. On the other hand, Anchor epitope residues are identical between P1, CA/04/09 and SI/03/06, suggesting P1 should be able to elicit broadly reactive antibodies targeting this conserved site.

### Glycosylation at COBRA P1 HA residue 127 impacts access to an antigenic site

**Fig. 4:**
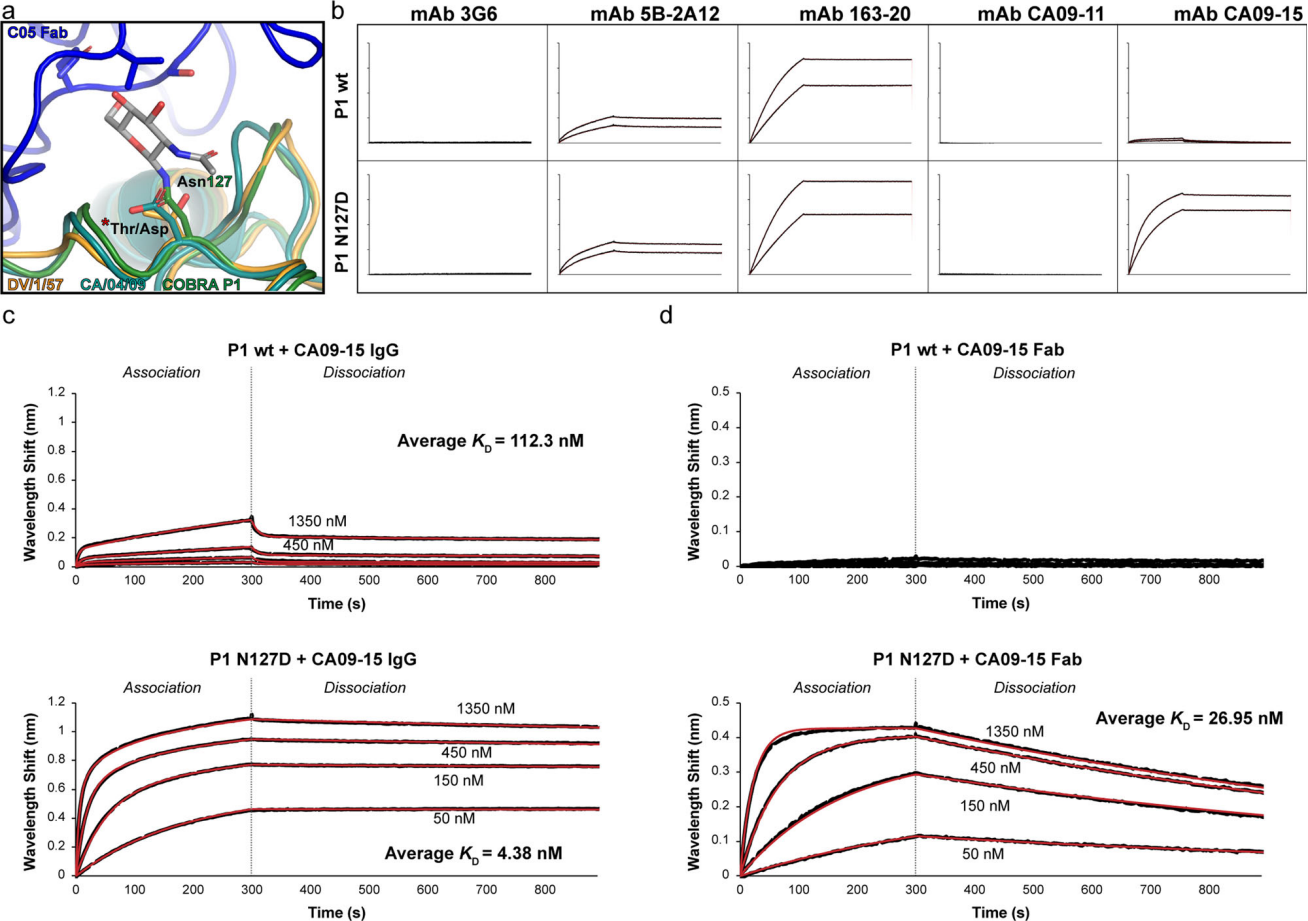
Impact of Asn127 glycosylation on antibody binding with COBRA P1. **a** Closeup of Asn127 of COBRA P1 overlayed with structures of CA/04/09 (PDB 3LZG) and DV/1/57 bound to the RBS-targeting antibody C05 (PDB 6ML8). **b** Biolayer interferometry of wildtype and N127D mutant COBRA P1 with antibodies known to target the head domain of CA/04/09 HA. **c** Kinetics of mAb CA09-15 binding with wildtype and mutant COBRA P1 measured by biolayer interferometry. Representative traces of the processed data are shown. The *K*_D_ value represents the mean of two independent experiments. **d** Kinetics of Fab CA09-15 binding with wildtype and mutant COBRA P1 measured by biolayer interferometry. Representative traces of the processed data are shown. The *K*_D_ value represents the mean of two independent experiments.

While analyzing the structure, we observed an unusual glycosylation site, Asn127, present in a hypervariable region of the HA protein (Fig. 1). Although not located within the classical antigenic sites, it is in close spatial proximity with Sa. Comparing the P1 structure to that of DV/1/57 HA bound to the RBS-targeting antibody C05 suggested that this glycosylation might present a steric hindrance to antibody binding (Fig. 4a). We hypothesized that glycosylation at this site in P1 could mask binding by some head-targeting antibodies. To test this, we generated a mutant P1 construct that deleted this glycosylation site by exchanging asparagine for aspartate, as is present in CA/04/09 (Figs. 1 and 4a). We then compared this mutant P1 (P1 N127D) with the unmodified construct (P1 wt) by measuring binding with a set of head-targeting monoclonal antibodies (mAbs) elicited by CA/04/09^11,19^ (Fig. 4b, Supplementary Fig. 4). Most of the mAbs exhibited similar binding properties for both the mutant and unmodified proteins, with the notable exception of CA09-15 which had a substantially higher affinity for P1 N127D compared to P1 wt. To gain a more precise measure of the difference in binding, we repeated the experiments with CA09-15 with expanded dilution series to quantify the affinity of binding (Fig. 4c, Supplementary Figs. 5 and 6). This revealed that CA09-15 mAb binds to P1 N127D with a 20 to 30-fold tighter affinity compared to P1 wt. Further characterization using the isolated fragment antigen-binding (Fab) region of CA09-15 showed even bigger differences (Fig. 4d and Supplementary Fig. 5). Whereas the monovalent Fab bound strongly to P1 N127D, no measurable binding occurring with P1 wt, suggesting that interactions with the wildtype antigen are primarily maintained through avidity. This suggests that the Asn127 glycosylation site may have a functionally impactful role on the repertoire of antibodies elicited by P1.

### Broadly reactive antibody 1F8 targets the RBS

**Fig. 5:**
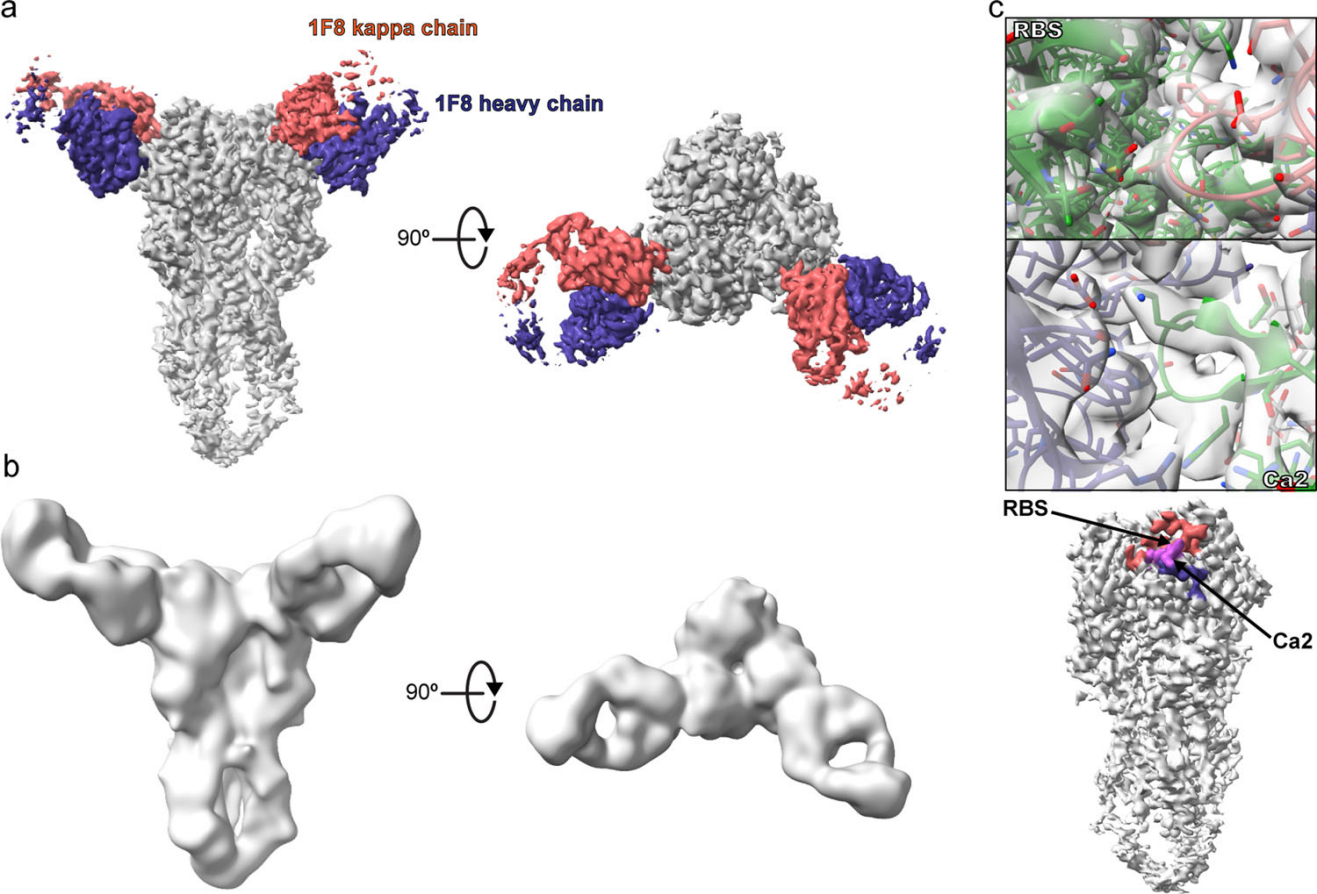
Cryo-EM structure of COBRA P1 bound to antibody 1F8. **a** 3D volume of the COBRA P1-1F8 complex, colored based on protein identity. **b** Gaussian filtered map (ChimeraX) clarifying the stem domain and constant regions of the Fab. **c** Closeup views of the epitope recognized by 1F8 (top) and overview of the antibody binding footprint (bottom). Regions of the footprint in HA that contact the light chain, heavy chain, or both are colored salmon, purple, or magenta, respectively.

The mAb 1F8, isolated from a P1-vaccinated mouse, is a broadly reactive antibody with an unknown epitope^11^. Specifically, mAb 1F8 displays broad hemagglutination inhibition activity against both historical seasonal and pandemic H1N1 influenza strains^11^. To elucidate the mechanism of broad reactivity, we performed cryo-EM studies of Fab 1F8 bound to P1 (Fig. 5, Supplementary Fig. 7, Table 2). The complex formed particles with diverse orientations as shown in the 2D classes, resulting in a 3D reconstruction with an average resolution of 3.1 Å (Supplementary Fig. 7). The refined volume consisted of two Fabs bound to a single trimer with well-resolved features between the antibody Fv regions and the HA head domain (Fig. 5a). This revealed 1F8, a high affinity antibody (see below), to bind a conformational epitope with a buried surface area of ~700 Å^2^. Examination of the regions of contact demonstrates that the epitope is comprised of the receptor binding site (RBS) and part of the Ca2 antigenic site (Fig. 5b). Notably, the light chain is the sole contributor to binding the RBS, while the heavy chain forms most of the interactions with the Ca2 antigenic site.

**Table 2:** Cryo-EM data collection, refinement and validation statistics.

	P1 COBRA + 1F8 Fab (EMD-26983) (PDB 8CT6)
Data collection and processing	
Magnification	130k
Voltage (kV)	300
Electron exposure (e^–^/Å^2^)	50.07
Defocus range (μm)	−0.8 to −1.8
Pixel size (Å)	1.045
Symmetry imposed	N/A
Initial particle images (no.)	111,535 (post 2D classification)
Final particle images (no.)	57,151
Map resolution (Å)	3.1
FSC threshold	0.143
Refinement	
Initial model used (PDB code)	7UYI
Model resolution (Å)	3.1
FSC threshold	0.143
Model composition	
Non-hydrogen atoms	14,918
Protein residues	1857
Ligands	23
*B* factors (Å^2^)	
Protein	86.59
Ligand	107.49
R.m.s. deviations	
Bond lengths (Å)	0.004
Bond angles (°)	0.590
Validation	
MolProbity score	1.77
Clashscore	12.75
Poor rotamers (%)	1.00
Ramachandran plot	
Favored (%)	97.16
Allowed (%)	2.84
Disallowed (%)	0.00

**Fig. 6:**
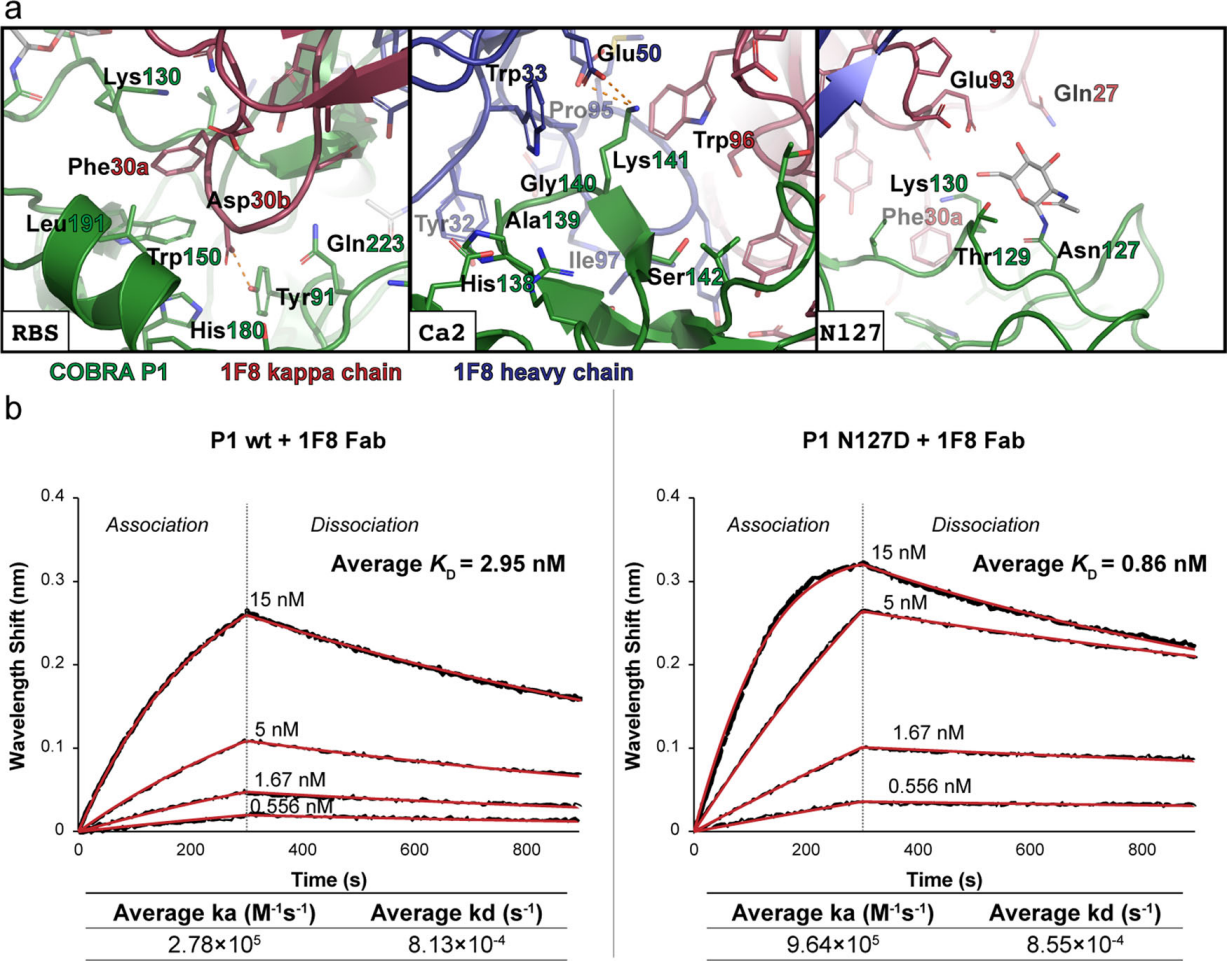
Molecular interactions between COBRA P1 and Fab 1F8. **a** Closeup views of the interface between COBRA P1 and 1F8 in the RBS, Ca2 antigenic site, and region near the Asn127 glycan. Electrostatic interactions are shown as orange dashes. **b** Biolayer interferometry kinetic assays of Fab 1F8 binding with wildtype and mutant COBRA P1. Representative runs of each are shown. The *K*_D_, rate of association (ka), and rate of dissociation (kd) values are reported as the mean of two independent experiments.

Looking more closely at the interface reveals the molecular details of the interactions that drive antibody binding (Fig. 6a). The complementary determining region loop 1 of the light chain (CDRL1) inserts into the RBS, with the major points of interaction occurring in Phe30a and Asp30b. This phenylalanine packs with Trp150, while other residues, including Lys130 and Leu191, form the edges of a hydrophobic pocket that facilitates this interaction. The aspartate forms an electrostatic interaction with Tyr91, with His180 and Gln223 in close enough proximity to potentially contribute additional transient interactions. At the Ca2 antigenic site, the CDR loops of the heavy chain and CDRL3 of the light chain frame a hydrophobic boundary around residues 138–142 of HA. Within this boundary, Glu50 in CDRH2 forms a salt bridge with Lys141 to seal the interaction. Interestingly, the sequence differences for both CA/04/09 and SI/3/06 HAs would remove this feature. In the case of CA/04/09, the lysine is shifted to the position of P1 residue Ser142. While there appears to be sufficient space to accommodate the larger side chain, the additional distance removes the potential to form the salt bridge. In SI/3/06, the substitution of the lysine for a glutamate introduces a direct charge repulsion between these residues that would be detrimental for binding. These structural features account for the previous observation that 1F8 has substantial, but lesser activity for CA/04/09 compared to P1, and much lower activity for SI/3/06^11^.

While examining the structure, we noticed that the glycosylation modification at Asn127 in P1 was in close proximity with Glu93 and Gln27 of the 1F8 light chain. To assess whether the glycosylation was contributing to antibody interaction, we measured the binding affinity of Fab 1F8 with both the P1 wt and N127D proteins (Fig. 6b). Both proteins bound 1F8 to a similar degree, with mean *K*_D_ values of 2.95 nM and 0.86 nM for the wildtype and mutant constructs, respectively. The small improvement in binding observed for the mutant can be attributed to a marginally faster association rate, suggesting that while glycosylation at residue 127 does not play a direct role in 1F8 binding, its absence results in a more readily accessible epitope.

### 1F8 is a unique RBS-targeting antibody

**Fig. 7:**
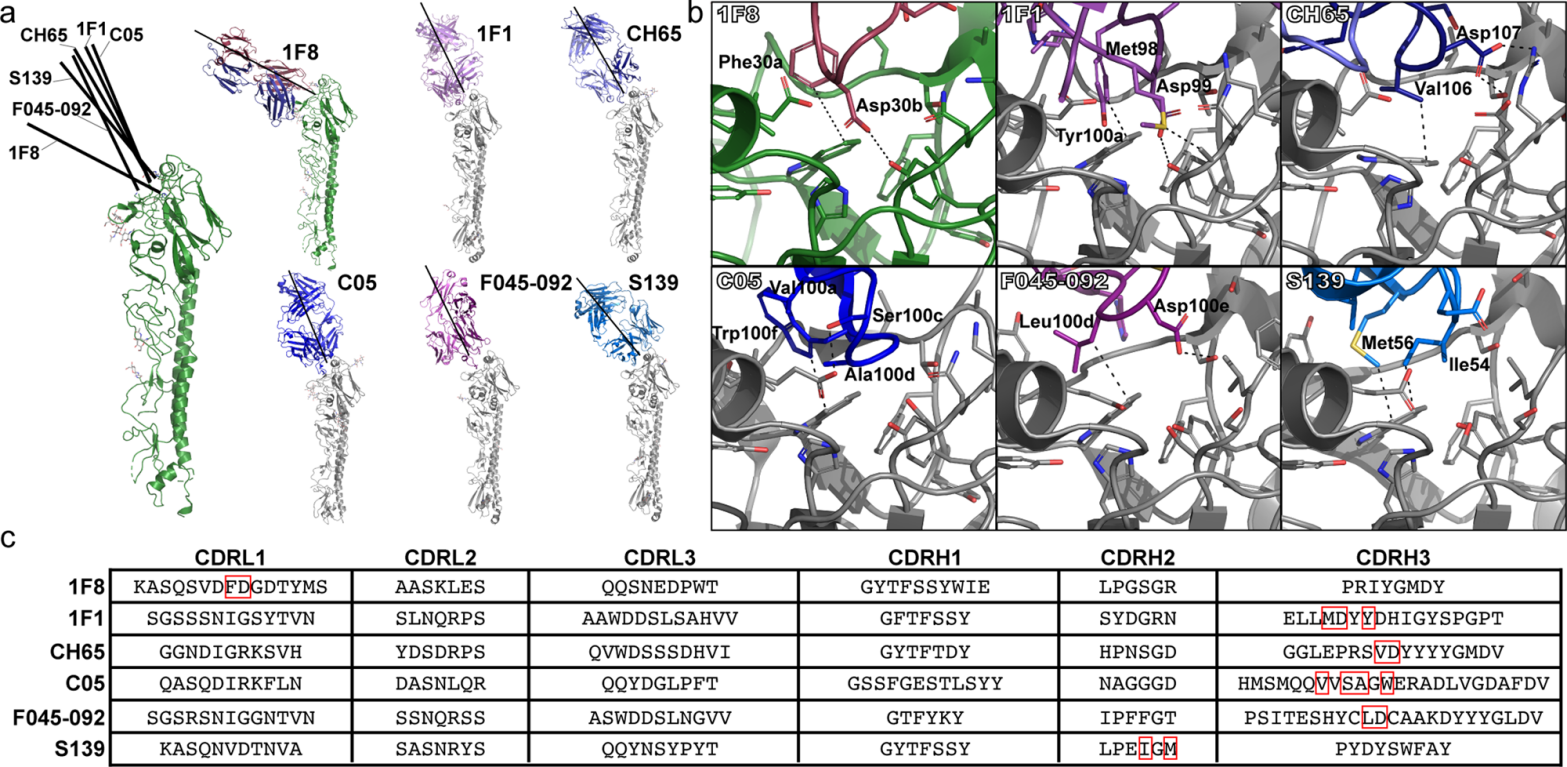
Comparison of 1F8 with other known RBS antibodies. **a** Overall view of the COBRA P1-1F8 mode of binding compared to antibodies 1F1 (PDB 4GXU), CH65 (PDB 5UGY), C05 (6ML8), F045-092 (PDB 4O58), and S139 (PDB 4GMS). Each structure is depicted as a single monomer bound to Fab, with line segments added to illustrate the relative orientations of the antibodies upon binding. **b** Closeup views of the RBS interactions for each antibody, with key contacts indicated by black dashed lines. **c** Complementarity Determining Regions of each antibody assigned according to the Chothia numbering system (annotated using the AbRSA server^80^). Residues forming important contacts within the RBS are boxed in red.

Antibodies that target the RBS have been a priority for the discovery and development of broadly reactive therapeutics^20–31^. To better understand the properties of 1F8, we compared our model to the known structures of other RBS antibodies bound to HA (Fig. 7). Looking at the general orientation of binding immediately reveals a stark contrast in how 1F8 engages HA compared to previously characterized antibodies (Fig. 7a). While most RBS antibodies approach from a steep, vertical angle of approach, 1F8 comes in almost perpendicular to the HA. Despite this difference, however, a closer examination of the interface reveals shared principles of binding in the RBS (Fig. 7b). In each case, the central feature is a hydrophobic residue packing with tryptophan in the RBS, supplemented with additional interactions. The antibodies 1F8, 1F1, CH65, and F045-092 contain a dipeptide motif, consisting of a hydrophobic residue followed by an aspartate, that is a common but non-universal feature of RBS antibodies^24,27–29^. In 1F8 and 1F1, the aspartate interacts with a universally conserved tyrosine, while in CH65 and F045-092 it forms electrostatic contacts with polar and/or charged residues along the edge of the RBS. While C05 lacks the dipeptide feature, it similarly forms polar interactions by means of a serine that interacts with a glutamate in HA. S139, though lacking any clear electrostatic contacts, has a strongly hydrophobic surface to drive packing with tryptophan.

Despite sharing common molecular features with other RBS antibodies, however, the way in which 1F8 accomplishes these interactions is vastly different. Most of the antibodies reach into the RBS by means of the heavy chain loops CDRH2 or CDRH3 (Fig. 7c). In contrast, the interaction in 1F8 is mediated by residues in the light chain loop CDRL1. The use of the light chain in part accounts for the different angle of binding and sets it apart from other RBS antibodies that have been structurally characterized to date.

## Discussion

Formulating influenza vaccines has long been a challenge due to the need to keep pace with an ever-changing landscape of circulating viruses. As new tools have emerged in vaccine technology, the prospects for developing more effective and durable vaccines have improved substantially, including the potential for “universal” vaccines to combat influenza. Structural characterization of candidate antigens plays a critical role for elucidating mechanisms of action and performing rational vaccine design.

In general, efforts to design more broadly protective influenza vaccines have sought to accentuate the conserved features of the HA protein. This includes methods to re-direct immune responses to the immunosubdominant stem domain, such as the use of “headless” HA, heterologous vaccination with different influenza subtypes, and chimeric HA containing mixed head-stem combinations^32–36^. Other attempts have engineered glycosylation sites to direct antibody responses away from the more variable regions^37–39^. The COBRA methodology is distinct from these approaches through its lack of specific predetermined antigenic targets within the HA. Rather, it relies on the input of sequence data from a target set of viruses to design a consensus that broadly represents those viruses, essentially predicting the protein of a hypothetical virus that bears traits of those within the design space. This presents two possible mechanisms that could account for the broad antibody responses observed for COBRA HA vaccines: (1) the elicitation of broadly reactive polyclonal antibody responses that target different epitopes in diverse viruses, or (2) the elicitation of broadly reactive antibodies that target conserved features. The structural and biochemical data presented here suggests that P1 may exhibit a degree of both.

The structural features of the classical head antigenic sites in COBRA P1 generally correlates with those of strains it is known to protect against. This is consistent with sequence-based analyses that have focused on residues in the vicinity of the Sa site^13^. Specifically, the presence or absence of Lys130 and glycosylation at residues 125 or 127 have been identified as “signatures” marking a divide between virus lineages in relation to the potency of antibody protection. Lys130 is present in pandemic-related sequences, including the 1918 and 2009 strains, whereas it is absent in many non-pandemic seasonal viruses such as SI/3/06. It structurally forms part of the outer boundary of the RBS, suggesting it could influence the way that RBS antibodies bind to block sialic acid receptor access^13^ (Fig. 6a). Glycosylation on residues 125 or 127 have the potential to shield epitopes in or around the Sa region of the HA head. Here we demonstrated that glycosylation on residue 127, specifically, interferes with the ability of antibody CA09-15 to bind P1.

Interestingly, the Asn127 glycosylation site present in P1 is a rare feature among circulating viruses. While this glycosylation site appears in a number of historic virus sequences, the most recent confirmed occurrence is the A/Mongolia/231/85 strain isolated in 1985^40^ (Supplementary Table 1). This glycan, along with several others, emerged among seasonal influenza strains in the decades following the 1918 pandemic. Compared to Asn125 glycosylation, which ultimately replaced it, the Asn127 glycan is more effective at evading polyclonal antibodies from mice immunized with pandemic H1N1 vaccines^40^. Conversely, immunization by a pandemic strain incorporating this glycosylation site resulted in more broadly reactive polyclonal antibody responses. When considered with other data showing similar impact of modifying vaccine glycosylation in H5N1^41^, this suggests that specific glycosylation sites in influenza hemagglutinin proteins may warrant particular attention for rational vaccine design.

Given the immune evasive properties of the Asn127 glycan, it may seem counterintuitive as to why it dropped out of circulating strains. There are a couple of potential factors that could play a role in the loss, and lack of re-acquisition, of this site. One is the level of sequence conservation inherent to this region of HA. Residues 127–130 tend to be variable across H1N1 virus sequences, whereas Asn125 and His126 are highly conserved across many strains^42^. From a statistical standpoint, this may present an advantage for glycosylation on Asn125 since only a single variable amino acid needs to fall into place to acquire the NXT motif, whereas residue 127 requires the coincidence of two or three variable amino acids to generate the motif. In addition, it is possible that there could be selective pressure against the predominance of the 127 glycan. Spatially, glycosylation at position 127 would be closer to the receptor binding site compared to Asn125. Although this likely provides more shielding against some neutralizing antibodies, a potential drawback could be less efficient access for receptor binding. This would be consistent with observations that HA head glycosylation sometimes impacts influenza virus infectivity^40,43,44^.

In the context of vaccine design, Asn127 in P1 highlights the ability of the COBRA methodology to incorporate even some less common structural features from a pool of input sequences, demonstrating the power to represent diverse viruses. Additionally, at a functional level this glycan shields a potentially variable epitope that results in more broadly protective polyclonal antibody responses, while also being permissive of access to the RBS by broadly neutralizing antibodies such as 1F8.

**Fig. 8:**
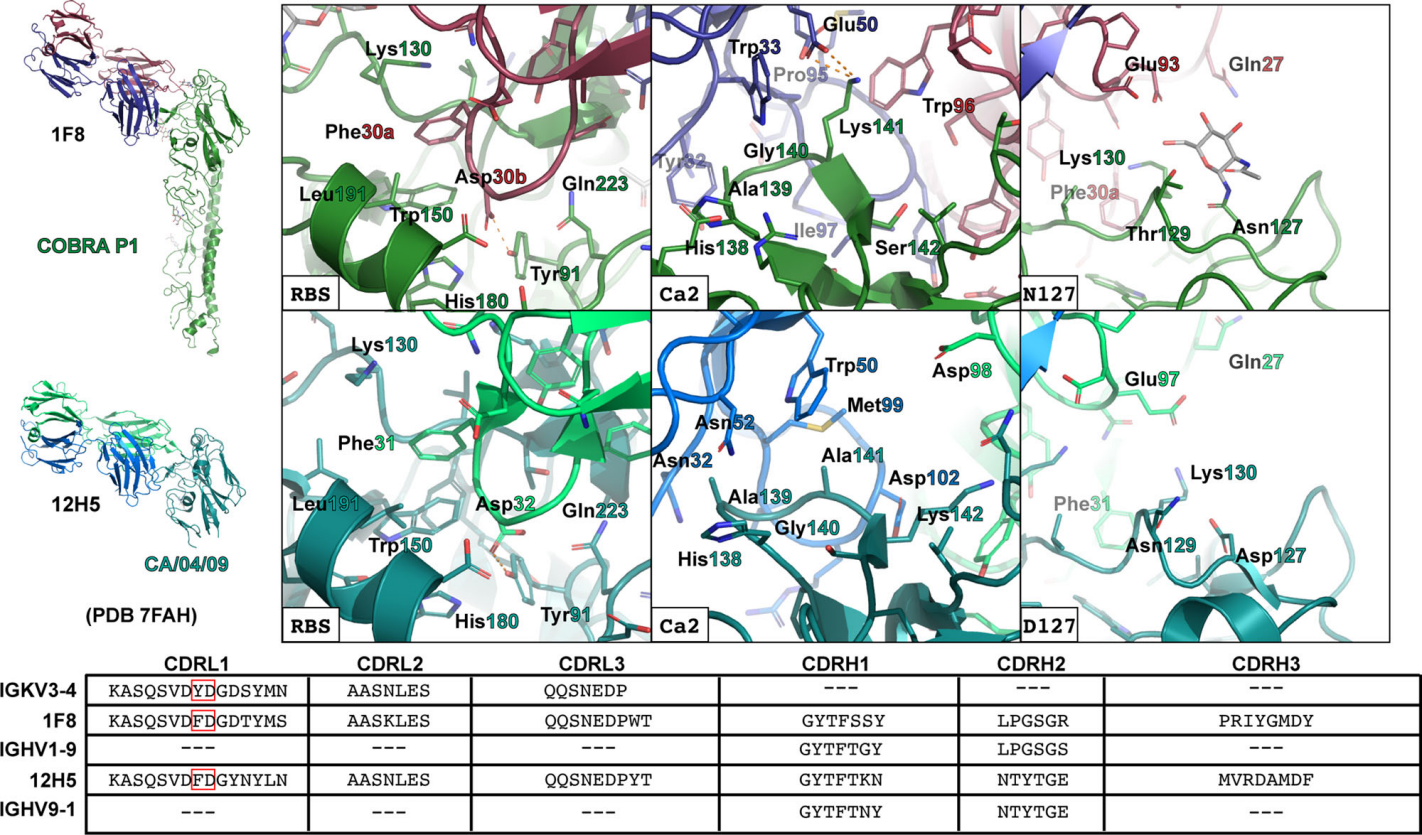
Comparison of 1F8 and 12H5 binding characteristics. The P1-1F8 and CA/04/09-12H5 (PDB entry 7FAH) structures were aligned based on the secondary structure structural alignment of the HA head domains. Overall structural representations are presented as cartoons of a monomeric HA bound to the Fab version of the antibody. Closeup views of the binding interfaces in the RBS, Ca2 antigenic site, and the region near residue 127 regions are depicted, with electrostatic interactions depicted by orange dashes. Comparisons of the CDR regions in germline antibody lineages versus antibodies 1F8 and 12H5 are shown.

1F8 stands out among structurally characterized RBS-targeting antibodies. The rare usage of CDRL1 to form the primary contact with the epitope positions the antibody in a distinct orientation from that of other RBS antibodies, resulting in a previously unobserved, atypical mode of binding. This engagement of the light chain with the RBS allows the heavy chain to interact with the Ca2 antigenic site to bolster the interaction. Prior to the discovery of 1F8, no antibodies from mice were reported in the literature that possessed both the same gene usage and reactivity profile. Although another antibody, 3D10, has been identified that utilizes the same VH germline gene, it lacks functional similarity to 1F8, being both non-neutralizing and having a narrower specificity^11^. The combined engagement achieved by 1F8 with the RBS and Ca2 accounts for both the broad reactivity and limitations in the breadth of binding^7^, as the RBS provides a conserved point of interaction while Ca2 in different HAs exhibits a range of favorable, tolerable, and conflicting levels of compatibility with 1F8. In principle, this mode of binding could allow for a constant usage of the light chain for RBS engagement that is paired with different heavy chains to optimize binding with Ca2. Interestingly, while this manuscript was in revision, a structure of another mouse-derived antibody, 12H5, was reported which shows a similar mode of binding with the head domain of CA/04/09 HA^45^ (Fig. 8). As predicted by our hypothesis, while sharing a common germline lineage for the light chain that results in a high degree of similarity to 1F8, 12H5 utilizes an alternate germline gene for the heavy chain that results in very different interactions with Ca2 that may be more optimal for binding with CA/04/09.

These two independent discoveries of a new class of broadly neutralizing antibody raises the question as to how these types of antibodies are elicited. Comparing the vaccination strategies utilized reveals contrasting approaches. While 1F8 was generated by a prime-boost regimen with a recombinant P1-expressing virus^11^, 12H5 was discovered after sequential immunization with three separate historical virus strains^45^. This has major implications in two ways. First, it emphasizes how the computationally designed P1, as a single antigen, elicits native antibody responses that are comparable with immunization from multiple wildtype strains. Second, it suggests that the generation of 1F8-like antibodies in mice may be a relatively common occurrence. This is supported by an analysis of antibody germline lineages, as searching for similar light chain sequences reveals close matches with the mouse germline genes IGKV3-4 and IGKV3-3, which possess YD and YN, respectively, in CDRL1 that aligns with the FD motif in 1F8 (Supplementary Table 2)^46^. Whether similar antibodies are present in human repertoires remains to be determined. Although no human light chain antibody sequences contain a chemically similar motif, with the closest match for the CDRL1, germline gene IGKV2-24, possessing SD at the corresponding amino acid positions, the FD motif could be generated by somatic hypermutation. Moreover, human antibody repertoires might be able to achieve a similar effect by means of an alternate mode of binding. Overall, the 1F8 structure sheds light on an additional means of attaining broad neutralization that could have applications for rational antibody design. It further emphasizes the effectiveness of computationally designed HA antigens, such as P1, in eliciting native antibody responses that are equivalent to heterologous immunization with wildtype antigens.

One potential limitation in evaluating the effectiveness of COBRA HA antigenicity stems from the fact that vaccine studies to this point have been restricted to preclinical animal models. While these have demonstrated the effectiveness of COBRA HA in both naïve and pre-immune models^10,47^, more data is needed to ascertain how it translates to human populations with more diverse genetic backgrounds and complex exposure histories. Recent studies have provided encouraging hints on this front, however, identifying broadly reactive antibodies in human vaccine cohorts that are cross-reactive with COBRA HAs^19,48^. This suggests that COBRA HAs retain structural elements capable of recalling existing protective antibody responses, while potentially broadening the immune response against additional strains.

The structural features observed with COBRA P1 naturally raises questions pertaining to optimal vaccine design. In what ways does it inform the future development of better antigens? One aspect highlighted by the structures is the distinction between sequence identity and structural similarity. Though related to each other, the sequence alone lacks clear spatial information to properly contextualize the impact of amino acid differences. This particularly applies to the composition of antigenic regions and the relative importance of individual residues in antibody binding interfaces. While sequence-based analysis has benefits from ease of use in a design pipeline, a more ideal approach is the incorporation of tertiary structural characteristics for more accurate measures of antigenic similarity. This includes the ability to ascertain the structural boundaries that demarcate between different lineages of viruses, such as seasonal viruses like SI/3/06 and pandemic-related such as CA/04/09, to understand the intrinsic limitations to the scope of protective antibodies that can be generated by a given antigen. In addition, the COBRA P1 structure demonstrates the ability to identify specific features, such as glycosylation at residue 127, that have a propensity to induce broader antibody responses and be of interest for deliberate incorporation in future generations of antigen design. Overall, the structural data enhances our understanding of the relationship between antigenic features and antibody protection and supports a paradigm of structure-based vaccine design.

In conclusion, the structures of COBRA P1 and 1F8 uncover crucial insights into the underlying molecular basis for the broad effectiveness of P1. This provides a framework to understand how COBRA HAs elicit protective antibodies against diverse viruses and the general effectiveness when compared to wildtype antigens. This further informs the rational antigen design process and progress towards the development of more effective next-generation influenza vaccines.

## Methods

### Expression and purification of COBRA P1 HA in insect cells

A codon-optimized synthetic gene encoding the COBRA P1 HA ectodomain was cloned into the pBacPAK8 vector (GenScript). A thrombin cleavage site, T4 fibritin Foldon trimerization domain, hexahistidine tag, and StrepTag II were encoded at the C-terminus. To ensure efficient secretion in the Sf9 baculovirus expression system, the gene was designed with a gp67 signal peptide sequence in place of the native secretion signal. The DNA plasmid was resuspended according to the manufacturer’s instructions and transformed into DH5α *E. coli* cells. A single colony was picked and used to inoculate a 5 mL LB overnight culture and subsequently midiprepped (Macherey-Nagel). Baculovirus was generated with the *flash*BAC™ system according to the manufacturer’s instructions (Mirus Bio). To induce expression, 2 L of Sf9 cells at a density of 3.8 × 10^6^ cells/mL were infected with ~23 mL of virus solution per liter of culture. The expression was continued for 4 days and the media harvested by centrifugation at 8000 × *g* for 20 min. The supernatant was sequentially filtered through glass microfiber, 0.45 μm, and 0.22 μm filters, then concentrated to approximately 150 mL by tangential flow with a VivaFlow® 200 (Sartorius). The sample was supplemented with 150 mM NaCl, 50 mM Tris [pH 8.0], and 1 mM EDTA (final concentrations), then centrifuged at 40,000 × *g* for 30 min. The clarified sample was filtered through a 0.22 μm filter and loaded onto two 5 mL StrepTrap columns connected in series (GE Healthcare). The columns were washed with 150 mM NaCl, 20 mM Tris [pH 8.0], 1 mM EDTA, and the protein was eluted with a gradient to 150 mM NaCl, 20 mM Tris [pH 8.0], 1 mM EDTA, 2.5 mM desthiobiotin on an ÄKTA Pure Chromatography system (GE Healthcare). The eluted protein was concentrated to approximately 4 mg/mL with a VivaSpin 50,000 MWCO concentrator, supplemented with glycerol to 5%, flash frozen in liquid nitrogen, and stored at −80 °C until further use.

### Crystallization and structural solution of COBRA P1 from insect cells

Frozen, affinity-purified P1 was thawed on ice. A thrombin digestion was performed overnight at 4 °C to remove the Foldon trimerization domain and tags. The digestion product was concentrated and loaded onto a Superdex 200 16/600 column to separate the HA and Foldon, as well as buffer exchange into 50 mM NaCl, 10 mM Tris [pH 7.5]. Fractions containing trimeric HA were pooled and concentrated to 10.2 mg/mL. The sample was screened with the MCSG Crystallization Suite (Anatrace) by sitting drop vapor diffusion using a Crystal Gryphon Robot (Art Robbins Instruments). Approximately 30 hits were observed 0–7 days after the initial screen. MCSG4 condition F4 (0.2 M MgCl_2_, 0.1 M Tris pH 8.5, 16% PEG 4000) successfully reproduced hexagonal-shaped crystals in manually set hanging drops that diffracted poorly (7–10 Å). The condition was further optimized using an Additive Screen (Hampton Research). Drops containing the additives PEG 400, Polypropylene glycol P 400, and Jeffamine M-600 pH 7 resulted in crystals with a different morphology (rectangular plates) that improved the diffraction. The final optimized condition contained 0.25 M MgCl_2_, 0.1 M Tris pH 8.5, 15% PEG 4000, 6% PEG 400. The crystal was cryoprotected in a solution consisting of 0.25 M MgCl_2_, 0.1 Tris pH 8.5, 15% PEG 4000, 6% ethylene glycol, 6% DMSO, 6% glycerol and subsequently flash cooled in liquid nitrogen. Diffraction data was collected using the JBluIce Graphical User Interface at the GM/CA beamline 23ID-D at the Advanced Photon Source. Data from a single crystal was processed in the CCP4 suite, using DIALS (version 2.2) for indexing and integration and AIMLESS for scaling and merging^49–51^. Molecular replacement was performed with Phaser in the PHENIX suite (version 1.19) using a computationally generated structure as the search model^52^. Six monomer copies were placed in the asymmetric unit, forming two intact trimers. The structure was refined with alternating rounds of manual and global building/refinement in COOT (version 0.9.8) and PHENIX^53,54^. The model was validated with Privateer and Molprobity^55,56^. Figures were made in PyMOL (version 2.3.0)^57^. The processed data and refined model have been deposited in the Protein Data Bank with accession number 7UYI. The residue numbering used in the model is based on the H1N1pdm numbering scheme^58^.

### Generation of the COBRA P1 N127D mutant

A plasmid encoding the COBRA P1 gene in vector pcDNA 3.1 (constructed as previously described^59^) was used as a template to generate the N127D mutant by site-directed mutagenesis (Thermo Fisher Scientific and New England Biolabs). Briefly, 5’ phosphorylated primers were designed to introduce a point mutation in the COBRA P1 (Integrated DNA Technologies). PCR reactions were set up with Phusion polymerase (NEB Biolabs) according to the Phusion site-directed mutagenesis protocol (Thermo Fisher Scientific). Following digestion with DpnI, the PCR product was circularized with T4 ligase (New England Biolabs) and transformed into DH5α *E. coli* cells. Mutated plasmid was miniprepped (Machery-Nagel) and confirmed by Sanger sequencing (GeneWiz).

### Expression and purification of COBRA P1 and COBRA P1 N127D for binding assays

The P1 wt and P1 N127D genes were cloned into a derivative of pcDNA3.1 optimized for protein expression in CHO cells^60^. The proteins were produced in CHO cells as previously described^61^. The harvested supernatants were supplemented with an approximately equivalent volume of Buffer A (500 mM NaCl, 20 mM NaH_2_PO_4_ [pH 8.0]), filtered, and loaded onto 5 mL HisTrap columns (GE Healthcare) equilibrated with Buffer A. The columns were washed with Buffer A, then the protein eluted with a gradient to Buffer B (500 mM NaCl, 20 mM NaH_2_PO_4_, 500 mM imidazole [pH 8.0]). The fractions were pooled, concentrated, and further purified by size exclusion chromatography on a Superdex 200 10/300 column (GE Healthcare) equilibrated in PBS. The presence and purity of the HA was confirmed by SDS-PAGE.

### Purification of monoclonal antibody CA09-11

The monoclonal antibody CA09-11 in mouse ascites was obtained through BEI Resources (Item# NR-28667). For purification, the sample was diluted in excess PBS and loaded onto a 5 mL Protein G HiTrap column (GE Healthcare). The column was washed with PBS and the protein eluted with 0.1 M Glycine-HCl [pH 2.03]. To neutralize the pH, 110 μL of 1.89 M Tris pH 8 was added to each 2 mL fraction. The fractions containing the peak were pooled, concentrated, and loaded onto a Superdex 200 10/300 column (GE Healthcare) equilibrated in PBS. The protein was confirmed by SDS-PAGE to check purity and quantified using an assumed extinction coefficient of 1.4 g/L and a 150 kD molecular weight.

### Purification of monoclonal antibody CA09-15 and Fab generation

The monoclonal antibody CA09-15 in mouse ascites was obtained through BEI Resources (Item# NR-28668). For purification, the sample was diluted in excess Protein A IgG Binding Buffer (Thermo Scientific) and added to a gravity flow column containing 0.5 mL of packed Protein A resin equilibrated with Binding Buffer. The column was washed with 3 mL of Binding Buffer, then the protein eluted with 3 mL of Protein A IgG Elution Buffer (Thermo Scientific) divided into three fractions. To neutralize the pH, 100 μL of 1 M Tris pH 8.5 was added to each 1 mL fraction. The presence of antibody was confirmed by SDS-PAGE, then the first two fractions combined and concentrated. CA09-15 mAb for BLI studies was further purified by size exclusion chromatography on a Superdex 200 10/300 column (GE Healthcare) equilibrated in PBS and quantified using an assumed extinction coefficient of 1.4 g/L and a 150 kD molecular weight.

Fab fragments were generated and purified using the Pierce™ Fab Preparation Kit according to the manufacturer’s instructions (Thermo Scientific) and the resulting product was further polished by size exclusion chromatography on a Superdex 200 10/300 column (GE Healthcare) equilibrated in PBS. The protein was quantified using an assumed extinction coefficient of 1.4 g/L and a 50 kD molecular weight.

### Expression and purification of Fab 1F8

The variable regions of the heavy (VH) and light (VL) chain sequence of 1F8 were determined as previously described^11^. In brief, total RNA was extracted from the corresponding 1F8 hybridoma cell line using the RNeasy MINI kit (Qiagen, Germantown, MD) according to the manufacturer’s instructions. RNA was reverse transcribed using the SuperScript III One-Step RT-PCR System (Thermo Fisher Scientific) and a pool of previously described primers^62^ according to the manufacturer’s instructions. Sequences of amplified VH and VL fragments were analyzed using the IMGT database^63^. Synthetic genes to produce recombinant Fab 1F8 were cloned into the pCI-Neo vector (GenScript). The variable domain of the light chain was designed in frame with a mouse kappa constant region, and the variable domain of the heavy chain with a mouse IgG1 CH1 domain containing a C-terminal thrombin cleavage site and TwinStrep tag. The protein was produced in CHO cells as previously described^61^. Post-expression, the media (~120 mL) was harvested and buffer adjusted with concentrated NaCl, Tris [pH 8.0], and EDTA to final concentrations of 150 mM, 50 mM, and 1 mM, respectively. To eliminate the impact of biotin in the media, 5 mL of BioLock (IBA Life Sciences) was mixed with the buffered media and filtered with a 0.22 μm filter. The sample was loaded onto a 5 mL StrepTrap column equilibrated with 150 mM NaCl, 20 mM Tris [pH 8.0], 1 mM EDTA. The column was washed, then the protein eluted with a 10 CV gradient to 150 mM NaCl, 20 mM Tris [pH 8.0], 1 mM EDTA, 6.5 mM desthiobiotin. The fractions containing the protein were thrombin digested overnight to remove the tag, then further purified using a Superdex 200 10/300 column equilibrated with 150 mM NaCl, 10 mM Tris [pH 7.5]. The fractions containing the Fab were pooled and concentrated, then flash frozen in liquid nitrogen and stored at −80 °C until use.

### Biolayer interferometry binding experiments

Biolayer interferometry data was collected on an Octet RED384 using the Data Acquisition Software (version 11.1.1.19). Binding experiments were performed in Assay Buffer (PBS, 1% BSA, 0.05% Tween 20). For initial binding studies, mAbs 3G6, 5B-2A12, 163-20 and CA09-11 and CA09-15 were diluted to 100 nM and 50 nM in assay buffer. Additional assays with the mAb and Fab versions of CA09-15 were performed with antibody concentrations ranging from 1350 nM to 50 nM. For Fab 1F8, assays were run with antibody concentrations ranging from 15 nM to 0.556 nM. All samples were run in duplicate (Supplementary Figs. 4–6, 8 and 9). Assays were performed with the temperature set to 30 °C and the plate shaking at 1000 rpm. To run the assays, anti-Penta-His biosensors soaked in Assay Buffer were equilibrated for 2 min in buffer. Subsequently, HA antigen at a nominal concentration of 10 μg/mL was loaded onto anti-Penta-His biosensors for 5 min. Following loading, the biosensors were returned to Assay Buffer for 2 min to obtain a baseline, then dipped into antibody samples for 5 min to measure association. Dissociation was measured by returning to Assay Buffer for 10 min. For mAb CA09-15, additional assays were performed loading the mAb onto AMC (anti-Mouse-Fc-capture) biosensors at 2 μg/mL for 5 min, with P1 wt or P1 N127D present as the analyte in concentrations ranging from 450 to 50 nM. Kinetics calculations were performed using the Octet Data Analysis HT software v7 (Sartorius). Each replicate was reference subtracted with a no antibody control, aligned to the baseline, and aligned to either the baseline or dissociation steps for inter-step correction. The initial assays with 3G6, 5B-2A12, 163-20, CA09-11 and CA09-15 as well as the full dilution assay of mAb CA09-15 with P1 N127D were fit using a 1:2 (bivalent analyte) binding model. The full assays with mAb CA09-15 and P1 wt fit best with a 2:1 (heterogenous ligand) model to account for the biphasic nature of dissociation, as well as the assays utilizing immobilized mAb CA09-15. Assays using Fab CA09-15 were fit with a 1:1 model of binding. For Fab 1F8, the data fit best using a 1:1 model with mass transport limitation due to the rapid association rates. The traces for each run were fit globally for curves in each dilution series. Model fits were evaluated and optimized based on visual inspection and the R^2^, χ^2^, and individual *K*_D_ error values. Average *K*_D_ values are reported as the mean of two independent experiments.

### Cryo-electron microscopy of the P1-1F8 complex

COBRA P1 used for electron microscopy studies was expressed and purified as described previously^59^. P1, 1F8, and the anchor antibody P1-05^19^ were combined in a 1:3:3 molar ratio and incubated at room temperature for 1 h to form complex. To freeze grids, octyl-β-glucoside (Anatrace) was added to samples at a final concentration of 0.1%. HA-Fab complex at 0.8 mg/mL was immediately deposited onto glow-discharged 1.2/1.3 quantifoil 400 mesh grids (EMS). The grids were blotted and frozen in liquid ethane using a Vitrobot (FEI), then transferred to liquid nitrogen for storage. Images were collected on a Titan Krios (FEI) with a Gatan K2 summit detector operating at 300 kV. A total of 1212 micrographs were collected in counting mode at a nominal magnification of 130,000, 1.045 Å/pixel, with Leginon^64^. The total exposure time was 6.8 s, with a total dose of 50.1 electrons/Å^2^. Data processing was performed with CRYOSPARC2^65^. Following motion correction and CTF estimation with GCTF^66^, particles were picked with the Cryosparc template picker. The particles were cleaned up by multiple rounds of 2D classification, followed by homogenous and heterogenous refinements. Due to the flexibility, poor reconstruction, and lack of specific interest in the details of Fab P1-05 for this manuscript, this antibody was masked out to focus on COBRA P1 and 1F8. Due to sub-stoichiometric quantities of bound Fab 1F8, the data was processed in C1 symmetry to preserve the overall quality of the two Fabs visible in the reconstruction. Multiple rounds of non-uniform, global CTF, and local CTF refinements yielded a final map with an overall resolution of 3.1 Å. Starting models were placed into the map in COOT. For COBRA P1, the X-ray crystal structure was used as the initial model. For 1F8, a model containing the Fv regions was generated with ROSIE^67–70^. Following model placement, alternating rounds of manual and global real space refinement were performed in COOT (version 0.9.8) and PHENIX (version 1.20)^71,72^. The model was validated in Molprobity, PHENIX, and EMRinger^56,73–75^. Figures were made in ChimeraX (version 1.2.5) and PyMOL (version 2.3.0)^57,76^. The map has been deposited in the Electron Microscopy Data Bank with accession number EMD-26983, and the refined model in the Protein Data Bank with accession number 8CT6.

### Reporting summary

Further information on research design is available in the Nature Portfolio Reporting Summary linked to this article.

## Supplementary information

Supplementary Information

Description of Additional Supplementary Data

Supplementary Data 1

Reporting Summary

## Data Availability

The processed data and model for the COBRA P1 X-ray crystal structure have been deposited with the Protein Data Bank (PDB; https://www.rcsb.org) as entry 7UYI. The processed cryo-EM map has been deposited with the Electron Microscopy Data Bank (EMDB; https://www.ebi.ac.uk/emdb/) as entry EMD-26983 and the structure model coordinates submitted to the PDB as entry 8CT6. Raw unprocessed data for biochemical assays are available upon reasonable request from the corresponding author. All other data are included in the main text and supplementary materials. Source data behind the biolayer interferometry graphs can be found in Supplementary Data 1.
