# Supplementary material for: Structural insights into the broad protection against H1 influenza viruses by a computationally optimized hemagglutinin vaccine
Source: Commun Biol. 2023 Apr 25;6:454. doi: 10.1038/s42003-023-04793-3 (PMC10126545; doi:10.1038/s42003-023-04793-3)
Supplement: Supplementary file 1 — Supplementary Information [file 42003_2023_4793_MOESM1_ESM.pdf]

Supplementary Information

Structural insights into the broad protection against H1 influenza viruses by a computationally optimized hemagglutinin vaccine

John V. Dzimianski[1], Julianna Han[2], Giuseppe A. Sautto[3], Sara M. O'Rourke[1], Joseph Cruz[1], Spencer R. Pierce[4], Jeffrey W. Ecker[4], Michael A. Carlock[4], Kaito A. Nagashima[4,5], Jarrod J. Mousa[4,5,6], Ted M. Ross[3,4,5], Andrew B. Ward[2], Rebecca M. DuBois[1]*

[1]*Department of Biomolecular Engineering, University of California Santa Cruz, Santa Cruz, California, USA*

[2]*Department of Integrative Structural and Computational Biology, The Scripps Research Institute, La Jolla, CA, USA*

[3]*Florida Research and Innovation Center, Cleveland Clinic, Port Saint Lucie, FL 34987, USA*

[4]*Center for Vaccines and Immunology, College of Veterinary Medicine, University of Georgia, Athens, GA, USA*

[5]*Department of Infectious Diseases, College of Veterinary Medicine, University of Georgia, Athens, GA, USA*

[6]*Department of Biochemistry and Molecular Biology, Franklin College of Arts and Sciences, University of Georgia, Athens, GA, USA*

*Corresponding author: rmdubois@ucsc.edu

This PDF includes:

Supplementary Figures 1 to 9

Supplementary Tables 1 and 2

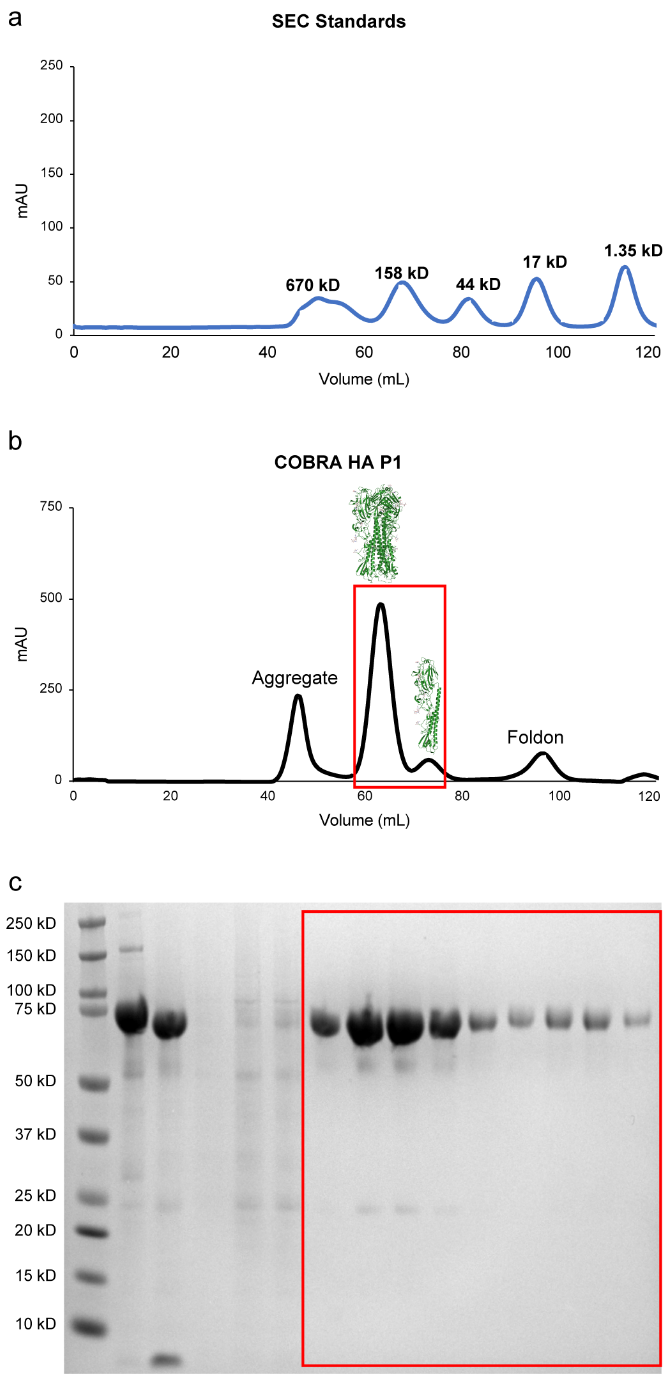

Supplementary Figure 1: Gel filtration of COBRA HA P1 for crystallography. (a) Molecular weight standards for size exclusion chromatography. (b) Size exclusion trace and (c) reducing SDS-PAGE gel of COBRA HA P1. Regions within the red boxes denote the fractions of interest.

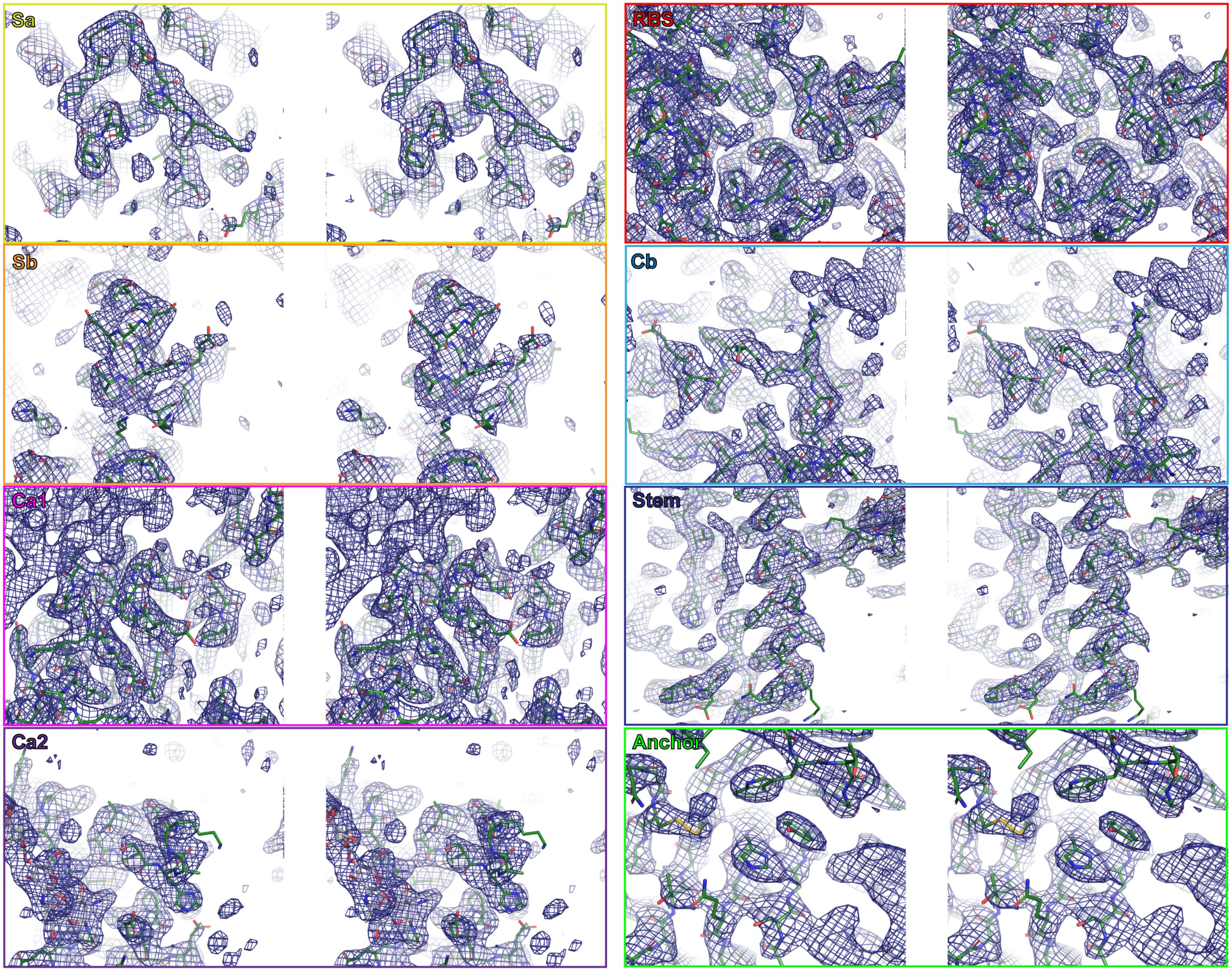
Sa
RBS
Sb
Cb
Ca1
Stem
Ca2
Anchor

Supplementary Figure 2: Electron density map of the COBRA P1. Wall-eye stereo views of the antigenic sites (Sa, Sb, Ca1, Ca2, Cb, Stem, and Anchor) and RBS of the COBRA P1 structure. A 2Fo-Fc map contoured at 1 sigma is shown for Chain A of the structure.

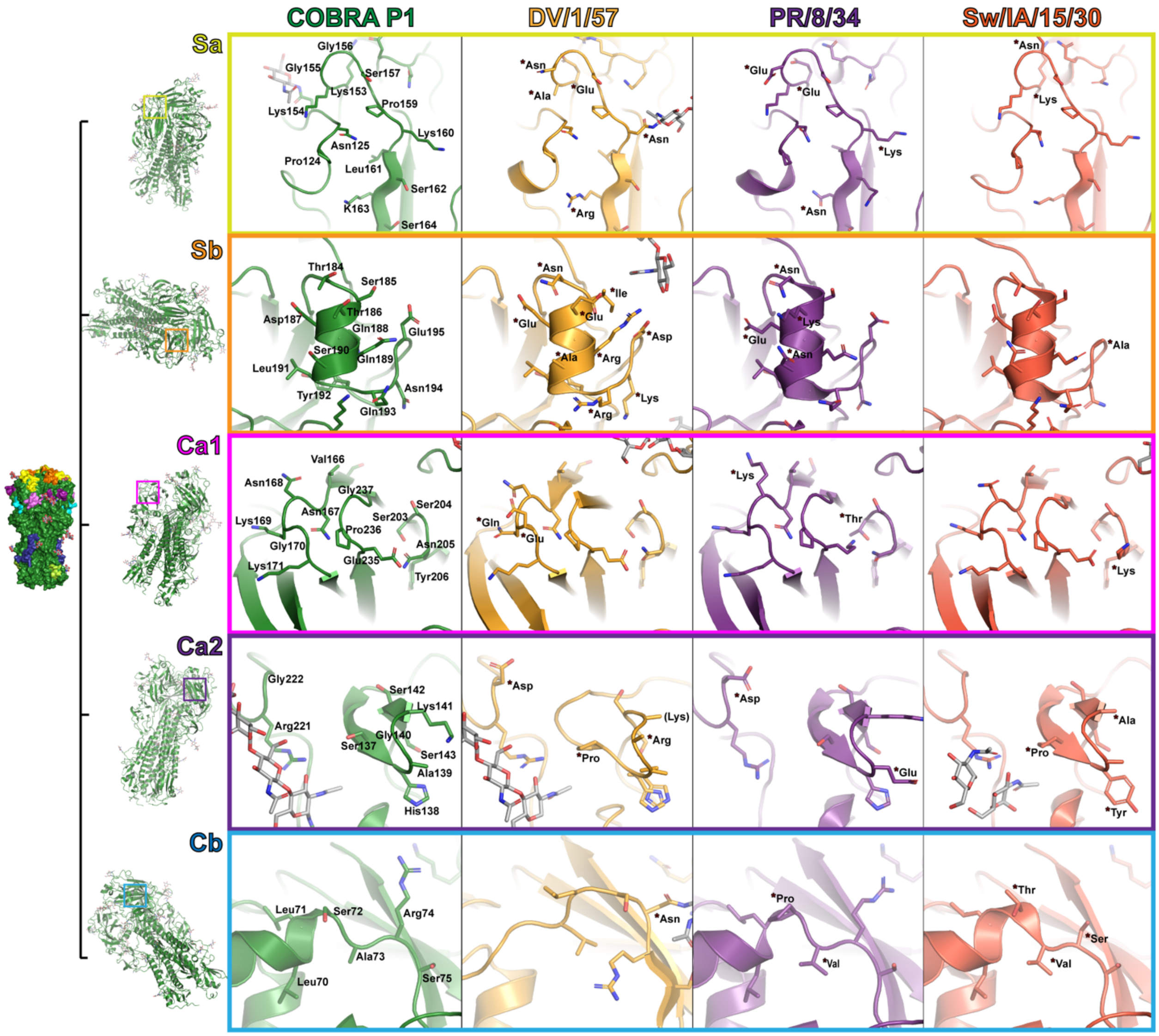

Supplementary Figure 3: Structural features of HA head antigenic sites. Crystal structures of HA from DV/1/57 (PDB 6ML8), PR/8/34 (PDB 1RU7) and Sw/IA/15/30 (PDB 1RUY) were overlayed on COBRA P1. The residues forming the major head antigenic sites Sa, Sb, Ca1, Ca2, and Cb are shown as sticks. Residue names and numbering are shown for P1 COBRA, with those that differ in the other HAs indicated by red asterisks with alternate amino acid labels.

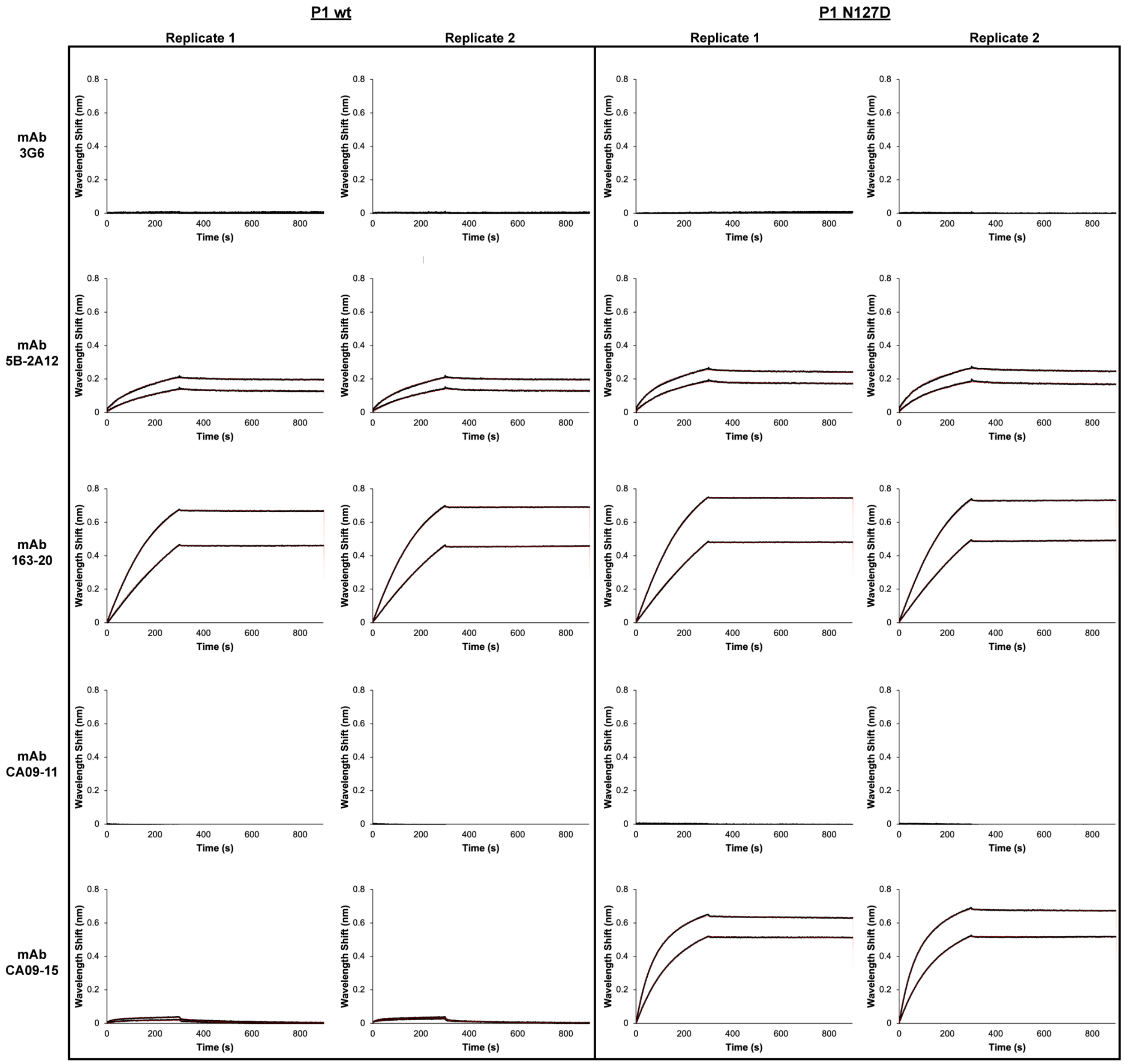

Supplementary Figure 4: Biolayer interferometry of P1 wt and P1 N127D with a panel of monoclonal antibodies. Replicate data is shown for the corrected traces in the association and dissociation phases for each binding experiment. The axes were scaled based on the antibody with the strongest binding.

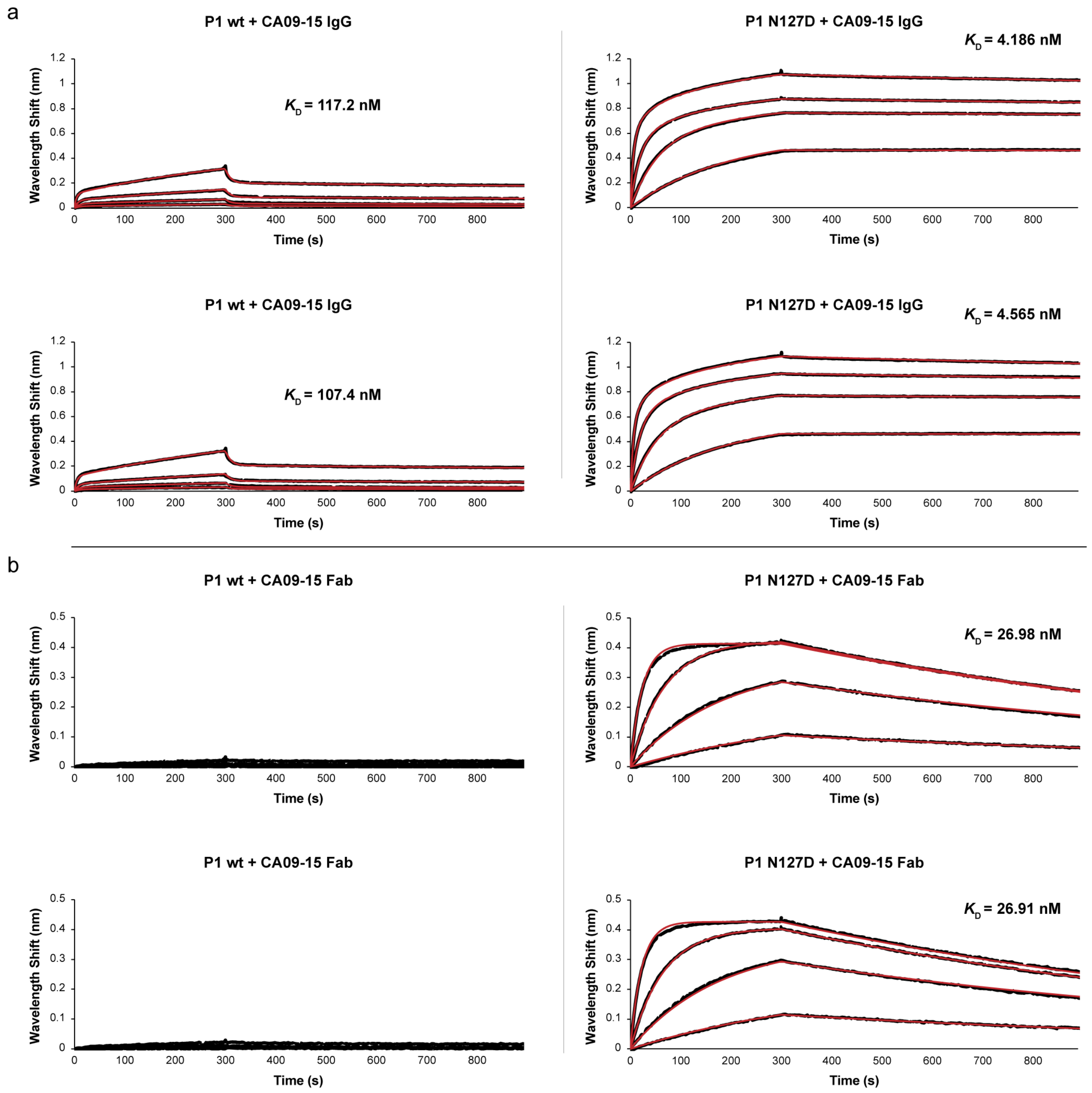

Supplementary Figure 5: Kinetic assays of P1 wt and P1 N127D with CA09-15. The corrected data traces for the association and dissociation phases are shown (black) with the associated model fits (red) for (a) mAb and (b) Fab binding with P1 wt and P1 N127D. The K_D values for each duplicate based on global fitting is reported.

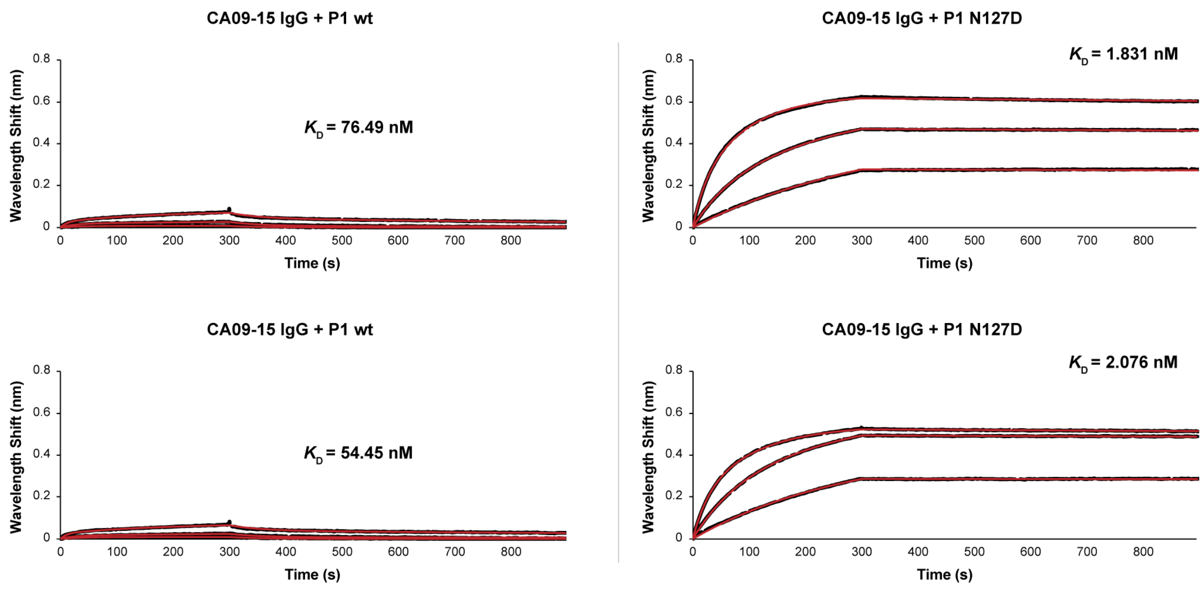

Supplementary Figure 6: Flipped assay orientation for mAb CA09-15 binding with P1 wt and P1 N127D. The corrected data traces for the association and dissociation phases are shown (black) with the associated model fits (red). The K_D values for each duplicate based on global fitting is reported.

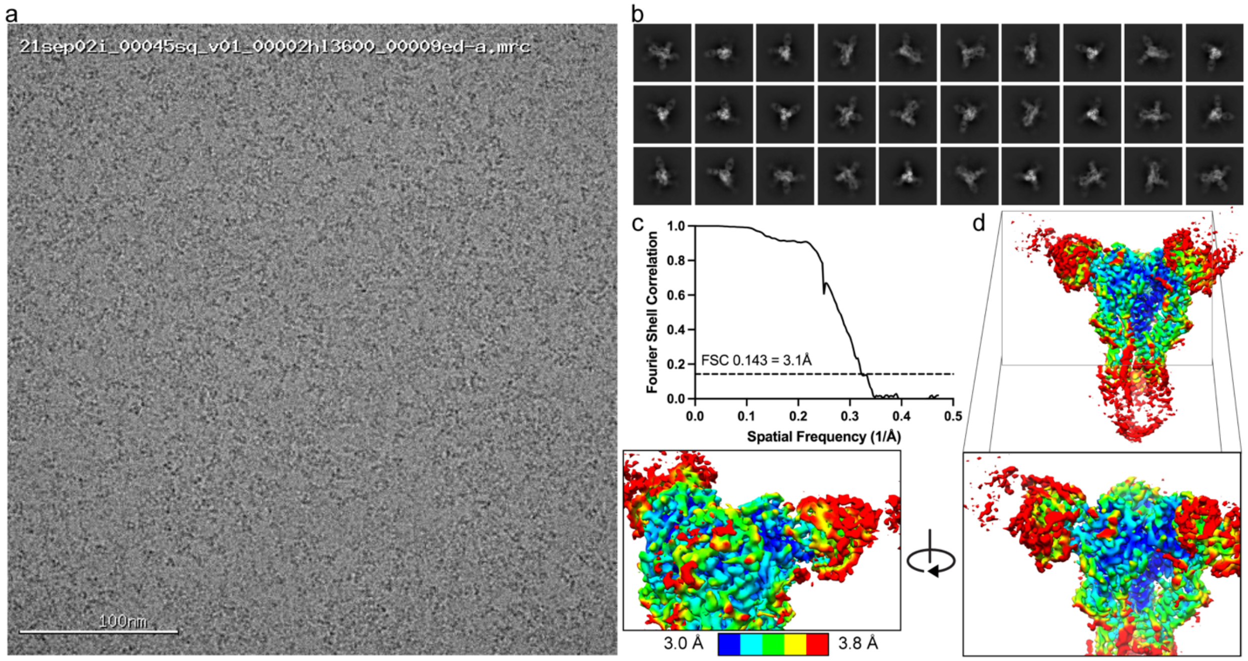

Supplementary Figure 7: Cryo-EM of the COBRA P1-1F8 Fab complex. (a) Representative micrograph containing HA-Fab particles. (b) 2D class averages of the antigen-antibody complex. (c) FSC curve for the refined map. (d) Local resolution map of the final refined 3D volume.

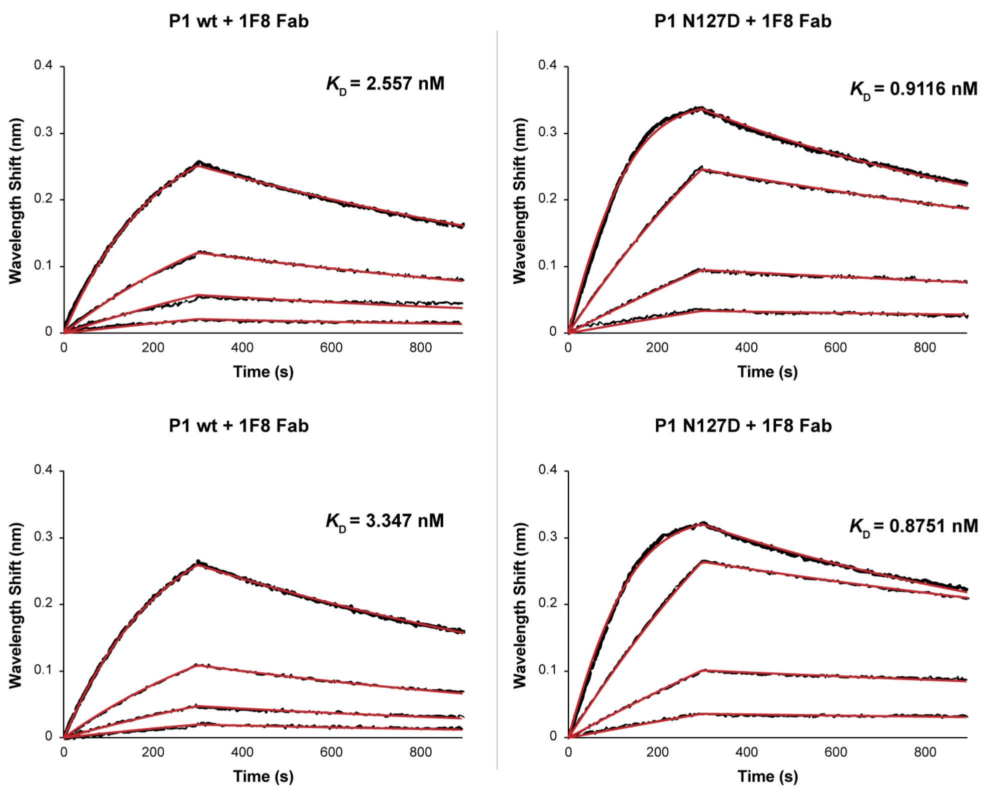

Supplementary Figure 8: Binding curves of 1F8 with P1 wt and P1 N127D. The corrected data traces for the association and dissociation phases are shown (black) with the associated model fits (red). The K_D values for each duplicate based on global fitting is reported.

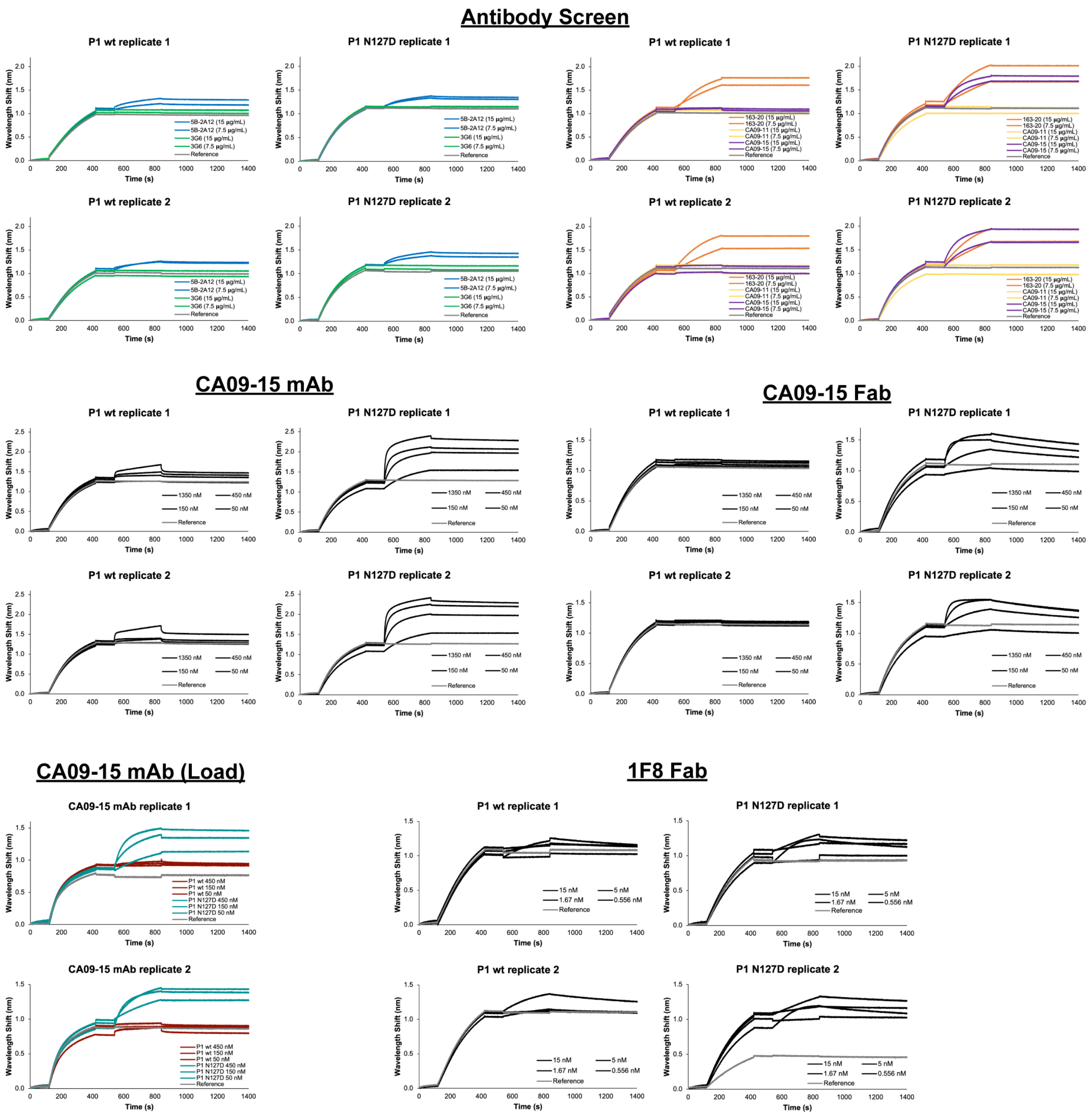

Supplementary Figure 9: Raw data traces from biolayer interferometry. Duplicate data for each assay is shown, with the reference biosensors in each experiment designated in gray.

Supplementary Table 1: Influenza H1N1 viruses with a glycosylation site at residue 127

Virus	Sequence (123-132; N-glycan motif)
A/Malaysia/54	WPKHNITRGV
A/USSR/90/1977	WPKHNVTRGV
A/Memphis/1/1978	WPKHNVTRGV
A/Memphis/10/1983	WPKHNVTKGV
A/Memphis/19/1983	WPKHNVTKGV
A/Albany/20/1978	WPKHNVTRGV
A/Memphis/27/1983	WPKHNVTKGV
A/Memphis/7/1980	WPKHNVTRGV
A/Memphis/20/1978	WPKHNVTRGV
A/Memphis/30/1983	WPKHNVTKGV
A/Memphis/28/1983	WPKHNVTKGV
A/Memphis/1/1984	WPNHNVTKGV
A/Memphis/1/1979	WPKHNVTRGV
A/Puerto Rico/8/1934	WPNHNTTKGV
A/AA/Marton/1943	WPKHNTTRGV
A/Memphis/49/1983	WPKHNVTRGV
A/Christ's Hospital/157/1982	WPNHNVTKGV
A/Phila/1935	WPKHNTTKGV
A/Memphis/48/1983	WPKHNVTKGV
A/Memphis/29/1983	WPKHNVTKGV
A/Memphis/26/1983	WPKHNVTKGV
A/Memphis/46/1983	WPKHNVTKGV
A/Maryland/2/1980	WPKHNVTRGV
A/Malaya/302/1954	WPKHNITRGV
A/Memphis/38/1983	WPKHNVTKGV
A/Arizona/14/1978	WPKHNVTRGV
A/Albany/12/1951	WPKHNITRGV
A/Brazil/11/1978	WPKHNITRGV
A/California/10/1978	WPKHNVTRGV
A/Albany/8/1979	WPKHNVTRGV
A/New Zealand/7/1983	WPNHNVTKGV
A/Memphis/39/1983	WPKHNVTKGV
A/Baylor/4052/1981	WPKHNVTRGV
A/Memphis/41/1983	WPKHNVTKGV
A/Chile/1/1983	WPKHNVTKGV
A/Memphis/14/1983	WPKHNVTKGV
A/Melbourne/1935	WPKHNTTKGV
A/Memphis/15/1983	WPKHNVTKGV
A/Memphis/18/1983	WPKHNVTKGV
A/Albany/4835/1948	WPKHNITRGV
A/Memphis/50/1983	WPKHNVTKGV
A/Memphis/2/1983	WPKHNVTRGV
A/Memphis/22/1983	WPKHNVTKGV
A/Memphis/15/1978	WPKHNVTRGV
A/Memphis/8/1983	WPKHNVTKGV
A/Memphis/11/1983	WPKHNVTKGV
A/Memphis/20/1983	WPKHNVTKGV
A/Memphis/13/1983	WPKHNVTKGV
A/California/45/1978	WPKHNVTRGV
A/Memphis/6/1983	WPKHNVTRGV
A/Memphis/1/1983	WPKHNVTRGV
A/Memphis/51/1983	WPKHNVTKGV
A/Memphis/47/1983	WPKHNVTKGV
A/Fort Monmouth/1/1947	WPKHNITRGV
A/Netherlands/001G1/1950	WPKHNITRGV
A/Netherlands/002P1/1951	WPKHNTTRGV
A/Netherlands/001R1/1953	WPKHNTTRGV
A/Fort Monmouth/1-MA/1947	WPKHNITRGV

A/USSR/90/1977	WPKHNVTRGV
A/Memphis/16/1983	WPKHNVTRGV
A/Fiji/15899/83	WPNHNVTKGV
A/CHR/157/83	WPNHNVTKGV
A/Leningrad/1954/1	WPKHNVTRGV
A/Chile/1/1983	WPKHNVTKGV
A/Kiev/59/1979	WPKHNVTRGV
A/Bellamy/JY2/1942	WPKHNTTKGV
A/Albany/14/1951	WPKHNITRGV
A/Liverpool/1951	WPKHNITRGV
A/Fort Monmouth/1-JY2/1947	WPKHNITRGV
A/Memphis/24/1983	WPKHNVTKGV
A/Albany/13/1951	WPKHNITRGV
A/USSR/46/1979	WPKHNVTRGV
A/India/6263/1980	WPKHNVTRGV
A/Memphis/44/1983	WPKHNVTKGV
A/Memphis/53/1983	WPKHNVTKGV
A/Memphis/35/1983	WPKHNVTKGV
A/USSR/90/1977	WPKHNVTRGV
A/Lackland/3/1978	WPKHNVTRGV
A/Tientsin/78/1977	WPKHNVTRGV
A/Memphis/3/1983	WPKHNVTKGV
A/Albany/4836/1950	WPKHNITRGV
A/Albany/1618/1951	WPKHNITRGV
A/Tonga/14/1984	WPNHNVTKGV
A/Memphis/21/1983	WPKHNVTKGV
A/Memphis/17/1983	WPKHNVTKGV
A/Memphis/23/1983	WPKHNVTKGV
A/Memphis/12/1983	WPKHNVTKGV
A/Memphis/4/1983	WPKHNVTKGV
A/AA/Huston/1945	WPKHNTTRGV
A/Memphis/40/1983	WPKHNVTKGV
A/Memphis/31/1983	WPKHNVTKGV
A/Bel/1942	WPKHNTTKGV
A/Memphis/10/1978	WPKHNVTRGV
A/Memphis/7/1983	WPKHNVTKGV
A/Memphis/25/1983	WPKHNVTKGV
A/Fort Monmouth/1/1947	WPKHNITRGV
A/Mongolia/231/85	WPKHNVTRGV
A/FLW/1951	WPKHNVTRGV
A/Melbourne/JY2/1935	WPKHNTTKGV
A/Puerto Rico/8-SV120/1934	WPDHNTTGVT
A/Roma/JY2/1949	WPKHNVTRGV
A/Malaysia/JY2/1954	WPKHNITRGV
A/Fort Monmouth/1/1947	WPKHNITRGV
A/Hong Kong/117/1977	WPKHNVTRGV
A/USSR/92/1977	WPKHNVTRGV
A/Memphis/42/1983	WPKHNVTKGV
A/Memphis/17/1978	WPKHNVTRGV
A/Memphis/32/1983	WPKHNVTKGV
A/Memphis/54/1983	WPKHNVTKGV
A/Roma/1949	WPKHNVTRGV
A/Memphis/11/1978	WPKHNVTRGV
A/Memphis/13/1978	WPKHNVTRGV

Supplementary Table 2: IgBLAST results with 1F8 and 12H5 CDRL1

Species	Antibody	CDRL1
Mouse	1F8	KASQS-VD<u>FD</u>GDTYMS
Mouse	12H5	KASQS-VD<u>FD</u>GYNYLN
Mouse	IGKV3-4	KASQS-VD<u>YD</u>GDSYMN
Mouse	IGKV3-3	RASQS-VD<u>YN</u>GISYMH
Mouse	IGKV1-135	KSSQS*L*LD<u>SD</u>GKTYLN
Human	IGKV2-24	RSSQS*L*VH<u>SD</u>GNTYLS

<u>Dipeptide</u>
Additional Residue
