# Supplementary material for: Structural insights into the broad protection against H1 influenza viruses by a computationally optimized hemagglutinin vaccine
Source: Commun Biol. 2023 Apr 25;6:454. doi: 10.1038/s42003-023-04793-3 (PMC10126545; doi:10.1038/s42003-023-04793-3)
Supplement: Supplementary file 2 — Description of Additional Supplementary Data [file 42003_2023_4793_MOESM2_ESM.docx]

**Description of Additional Supplementary Files**

**File name:** Supplementary Data 1

**Description:** Source data behind the biolayer interferometry graphs and calculated kinetic values in Figs. 3,4, and 6 and Supplementary Figs. 5,6, and 8.
