# Supplementary material for: Structural insights into the broad protection against H1 influenza viruses by a computationally optimized hemagglutinin vaccine
Source: Commun Biol. 2023 Apr 25;6:454. doi: 10.1038/s42003-023-04793-3 (PMC10126545; doi:10.1038/s42003-023-04793-3)
Supplement: Supplementary file 4 — Reporting Summary [file 42003_2023_4793_MOESM4_ESM.pdf]

nature portfolio

Corresponding author(s): Rebecca M. DuBois

Last updated by author(s): 03/27/2023

Reporting Summary

Nature Portfolio wishes to improve the reproducibility of the work that we publish. This form provides structure for consistency and transparency in reporting. For further information on Nature Portfolio policies, see our Editorial Policies and the Editorial Policy Checklist.

Statistics

For all statistical analyses, confirm that the following items are present in the figure legend, table legend, main text, or Methods section.

n/a	Confirmed	
☐	☒	The exact sample size (n) for each experimental group/condition, given as a discrete number and unit of measurement
☐	☒	A statement on whether measurements were taken from distinct samples or whether the same sample was measured repeatedly
☒	☐	The statistical test(s) used AND whether they are one- or two-sided<br>*Only common tests should be described solely by name; describe more complex techniques in the Methods section.*
☒	☐	A description of all covariates tested
☒	☐	A description of any assumptions or corrections, such as tests of normality and adjustment for multiple comparisons
☐	☒	A full description of the statistical parameters including central tendency (e.g. means) or other basic estimates (e.g. regression coefficient) AND variation (e.g. standard deviation) or associated estimates of uncertainty (e.g. confidence intervals)
☒	☐	For null hypothesis testing, the test statistic (e.g. F, t, r) with confidence intervals, effect sizes, degrees of freedom and P value noted<br>*Give P values as exact values whenever suitable.*
☒	☐	For Bayesian analysis, information on the choice of priors and Markov chain Monte Carlo settings
☒	☐	For hierarchical and complex designs, identification of the appropriate level for tests and full reporting of outcomes
☒	☐	Estimates of effect sizes (e.g. Cohen's d, Pearson's r), indicating how they were calculated

Our web collection on statistics for biologists contains articles on many of the points above.

Software and code

Policy information about availability of computer code

Data collection	Crystal X-ray diffraction data were collected using the JBluIce Graphical User Interface at GM/CA at APS. Cryo-EM data was collected using Leginon. Biolayer interferometry data was collected on an Octet RED384 using the Data Acquisition Software (version 11.1.1.19).
Data analysis	Crystal data was processed and analyzed in DIALS (version 2.2), AIMLESS, PHENIX (version 1.19), Coot (version 0.9.8), and PyMOL (version 2.3.0). Cryo-EM data was processed an analyzed in CryoSPARC2, ChimeraX (version 1.2.5), Coot (version 0.9.8), PyMOL (version 2.3.0), and PHENIX (version 1.20). Biolayer interferometry data was processed and analyzed in the Octet RED384 Data Analysis HT software (version 11.1.1.39).

For manuscripts utilizing custom algorithms or software that are central to the research but not yet described in published literature, software must be made available to editors and reviewers. We strongly encourage code deposition in a community repository (e.g. GitHub). See the Nature Portfolio guidelines for submitting code & software for further information.

Data

Policy information about availability of data

All manuscripts must include a data availability statement. This statement should provide the following information, where applicable:

- Accession codes, unique identifiers, or web links for publicly available datasets
- A description of any restrictions on data availability
- For clinical datasets or third party data, please ensure that the statement adheres to our policy

The processed data and model for the COBRA P1 X-ray crystal structure have been deposited with the Protein Data Bank (PDB; https://www.rcsb.org) as entry 7UYI. The processed cryo-EM map has been deposited with the Electron Microscopy Data Bank (EMDB; https://www.ebi.ac.uk/emdb/) as entry EMD-26983 and the structure model coordinates submitted to the PDB as entry 8CT6. Raw data for biochemical assays are available upon request from the corresponding author.

Human research participants

Policy information about studies involving human research participants and Sex and Gender in Research.

Reporting on sex and gender	Sex and gender were not considered in the study design.
Population characteristics	No human research participants were included on the study.
Recruitment	See above
Ethics oversight	See above

Note that full information on the approval of the study protocol must also be provided in the manuscript.

Field-specific reporting

Please select the one below that is the best fit for your research. If you are not sure, read the appropriate sections before making your selection.

☒ Life sciences ☐ Behavioural & social sciences ☐ Ecological, evolutionary & environmental sciences

For a reference copy of the document with all sections, see nature.com/documents/nr-reporting-summary-flat.pdf

Life sciences study design

All studies must disclose on these points even when the disclosure is negative.

Sample size	Only in vitro assays on isolated macromolecules were performed, rendering sample size calculations unnecessary. Assays were performed in duplicate to ensure replication of the results.
Data exclusions	Outliers and weak/low quality data obtained from X-ray diffraction and Cryo-EM were excluded according to standard practices. Some data from biolayer interferometry experiments were excluded as outliers based on instrument limits of detection and/or poor fit to the kinetic model.
Replication	Antibody binding experiments were performed in duplicate and successfully replicated.
Randomization	Not relevant to study--In vitro binding assays of each antibody was performed across all experimental groups.
Blinding	Not relevant to study--no randomization of group allocation.

Reporting for specific materials, systems and methods

We require information from authors about some types of materials, experimental systems and methods used in many studies. Here, indicate whether each material, system or method listed is relevant to your study. If you are not sure if a list item applies to your research, read the appropriate section before selecting a response.

Materials & experimental systems

n/a	Involved in the study
☐	☒ Antibodies
☐	☒ Eukaryotic cell lines
☒	☐ Palaeontology and archaeology
☒	☐ Animals and other organisms
☒	☐ Clinical data
☒	☐ Dual use research of concern

Methods

n/a	Involved in the study
☒	☐ ChIP-seq
☒	☐ Flow cytometry
☒	☐ MRI-based neuroimaging

Antibodies

Antibodies used

Antibodies 1F8, P1-05, 3G6, 5B-2A12, and 163-20 were generated in-house. Antibodies CA09-15 and CA09-11 were obtained through BEI Resources, NIAID, NIH, item numbers NR-28668 and NR-28667, respectively.

Validation

Reactivity of CA09-15 and CA09-11 are certified by BEI Resources. Reactivity of antibodies 1F8, 3G6, and 5B-2A12 are demonstrated in Sautto et al. 2020 (DOI: https://doi.org/10.4049/immunohorizons.1800044) and the present manuscript (Figures 4b, 4c, 5, and 6b). Reactivity of antibodies P1-05 and 163-20 are shown in Nagashima et al. 2021 (DOI: https://doi.org/10.1101/2021.10.25.465669), as well as the present manuscript for antibody 163-20 (Figure 4b).

Eukaryotic cell lines

Policy information about cell lines and Sex and Gender in Research

Cell line source(s)

Standard cell lines were obtained from Expression Systems (Sf9) and Thermo Fisher Scientific (EXPI293F, Frestyle 293F, and CHO-S).

Authentication

None of the cell lines used were authenticated.

Mycoplasma contamination

Cell lines were not tested for Mycoplasma contamination.

Commonly misidentified lines
(See ICLAC register)

Name any commonly misidentified cell lines used in the study and provide a rationale for their use.
